# Eastern African traditional fermented foods and beverages: Advancements, challenges, and perspectives on food technology, nutrition, and safety

**DOI:** 10.1111/1541-4337.70137

**Published:** 2025-03-04

**Authors:** Habtamu Hawaz, Benedetta Bottari, Francesca Scazzina, Eleonora Carini

**Affiliations:** ^1^ School of Animal and Range Sciences, College of Agriculture Hawassa University Hawassa Ethiopia; ^2^ Department of Food and Drug University of Parma Parma Italy

**Keywords:** beverages, Eastern Africa, fermentation, milk, traditional food

## Abstract

Traditional fermented foods and beverages have played a vital role in the diet, culture, and economy of Eastern African countries for centuries, contributing significantly to food security, poverty alleviation, and sustainable development. Despite their importance, comprehensive documentation of their production methods, nutritional benefits, and safety challenges remains limited. This review critically examines the most widely consumed fermented foods and beverages in the region, derived from both plant and dairy sources, with a focus on their processing technologies, microbial dynamics, nutritional profiles, and food safety issues. Data were gathered from a systematic review of published and unpublished scientific research between March and April 2023. These products are predominantly obtained through spontaneous fermentation, a sustainable bioprocessing method that enhances shelf life, nutritional value, and sensory attributes. A diverse range of products, including non‐alcoholic and alcoholic beverages, porridges, breads, and yogurt‐like dairy products, rely heavily on the activity of lactic acid bacteria and yeasts. While these foods are rich in essential nutrients such as carbohydrates, proteins, vitamins, and minerals, the non‐standardized fermentation processes often result in inconsistent quality and pose risks related to foodborne pathogens and toxins. This review emphasizes the urgent need for developing standardized fermentation practices, including the isolation and application of starter cultures, to improve safety and product quality. Furthermore, scaling up traditional fermentation methods for commercialization offers significant opportunities to enhance regional nutrition and economic development while addressing the challenges of food safety and quality assurance.

## 1 INTRODUCTION

Traditional fermented foods and beverages have been a cornerstone of Eastern Africa's culinary, cultural, and economic landscape for centuries. Several kinds of raw materials, such as milk and meat, starchy root crops, vegetables, and cereal grains (i.e., maize, sorghum, millet, and wheat), are fermented to obtain foods, while beverages are predominantly made from cereals (Johansen et al., 2019). The Eastern African region faces significant challenges regarding food insecurity and foodborne illnesses. According to the Food and Agriculture Organization (FAO, 2023), nearly 30% of the population in the region is undernourished, with an alarming prevalence of chronic malnutrition among children under 5 years old. Despite the production of various food crops and animal products, food insecurity remains significantly higher in the Eastern African region, compared to other parts of the world. In recent years, especially during the 2021–2022 drought, there has been an increase in the number and percentage of undernourished individuals, as well as those experiencing moderate and severe food insecurity (Omay et al., 2024). A study conducted by G. G. Gebre (2021) found that 15% of households in the region were mildly food‐secure, 26% were moderately food‐insecure, and 7% were severely food‐insecure. Furthermore, unsafe food practices and limited access to clean water exacerbate the incidence of foodborne illnesses, which disproportionately affect vulnerable groups such as children and the elderly. Data from the World Health Organization (WHO, 2025) indicate that common foodborne pathogens, including *Salmonella, Escherichia coli*, and *Listeria monocytogenes*, are major contributors to health issues, leading to both acute and long‐term consequences for affected populations. Addressing these issues requires a multi‐faceted approach, including improvements in food processing, preservation methods, and education on food safety. Fermented foods, with their potential to improve food safety through microbial competition and production of antimicrobial compounds, offer a promising avenue for reducing foodborne illness and enhancing food security in the region. By understanding the dual challenges of food insecurity and foodborne illness, the importance of promoting and standardizing traditional fermentation practices becomes evident. This context also underscores the role fermented foods can play in improving regional nutrition and public health outcomes.

**FIGURE 1:**
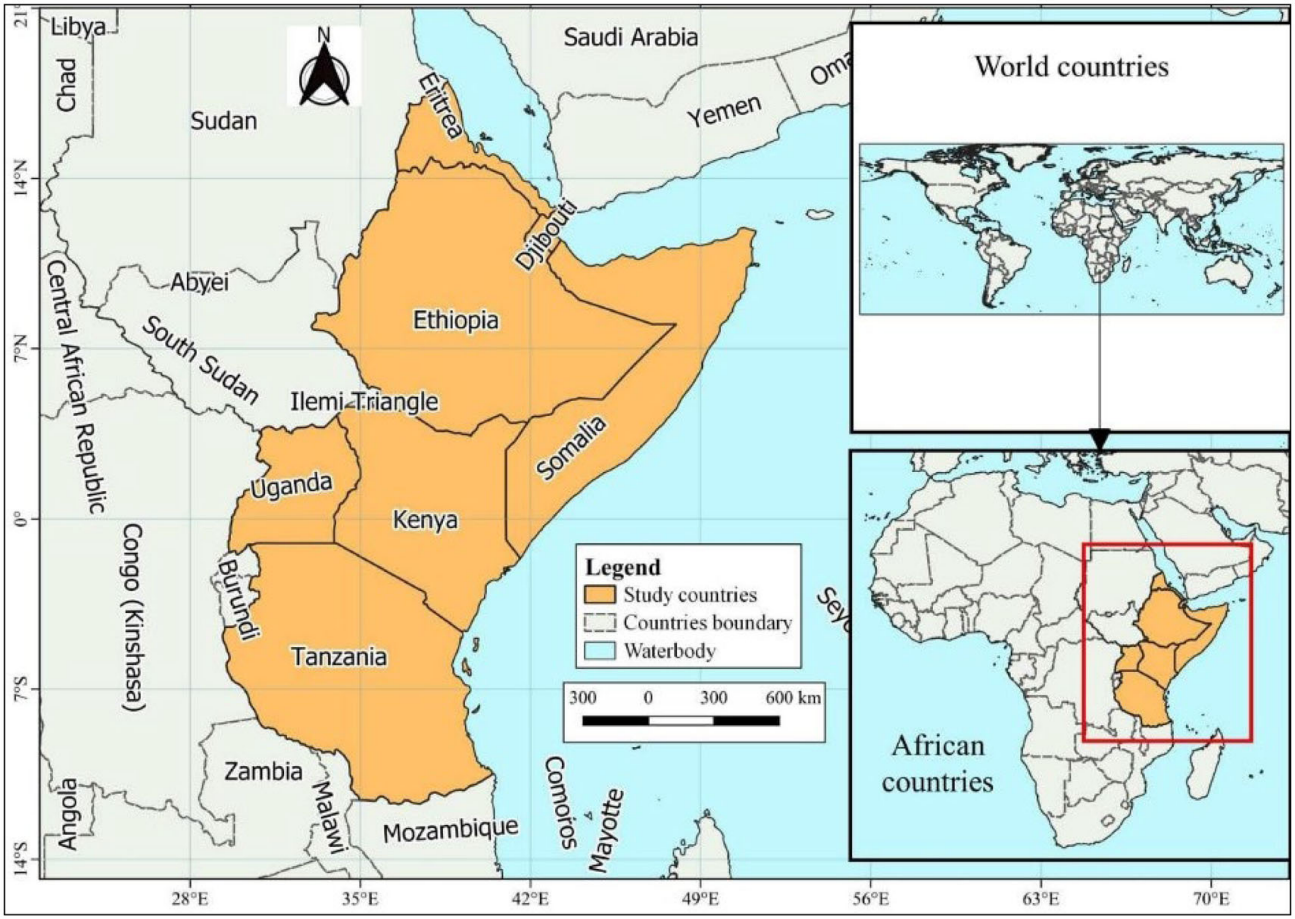
Map of the Eastern African region: East Africa and the Horn of Africa.

**FIGURE 2:**
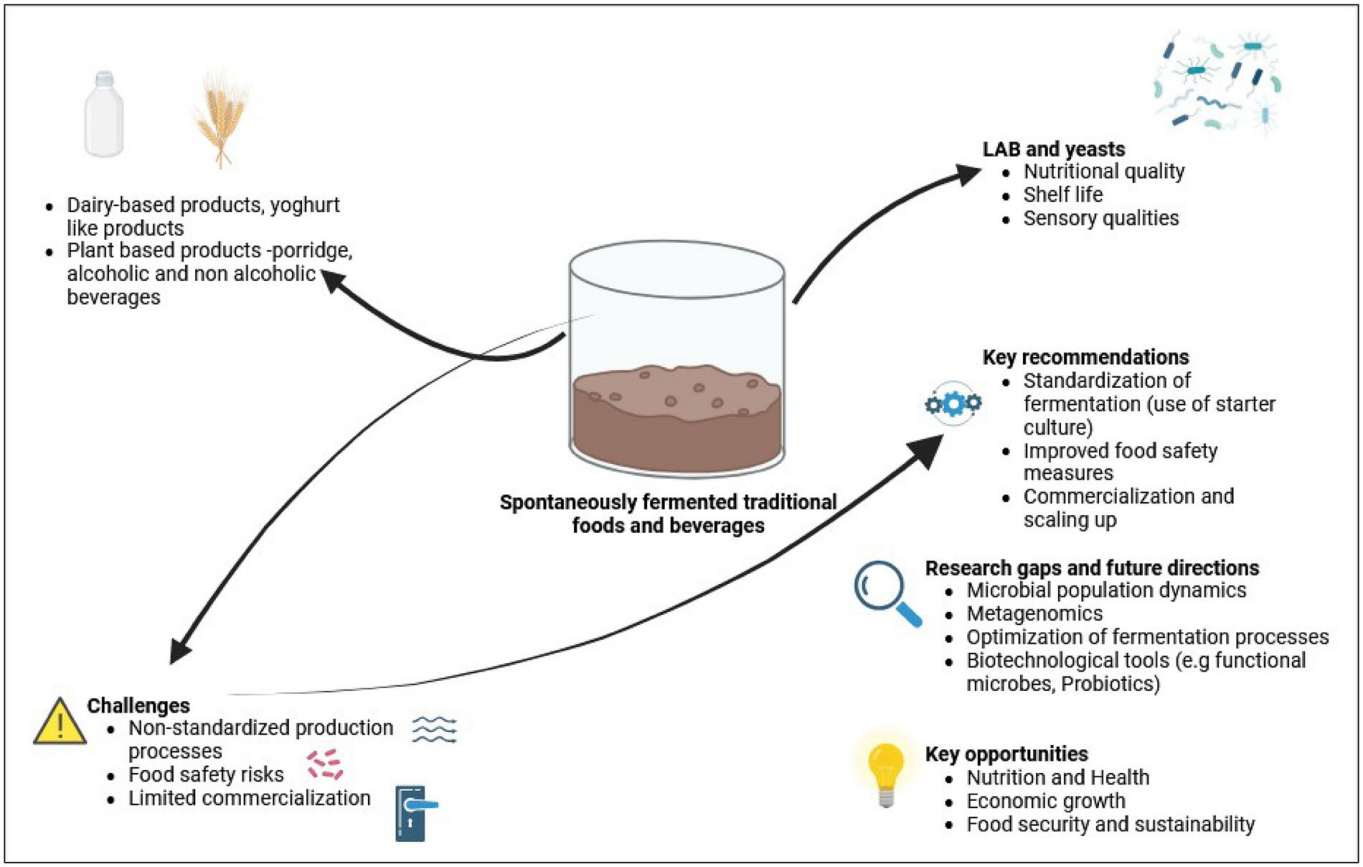
Key insights and contributions of Eastern African traditional fermented foods and beverages. LAB, lactic acid bacteria.

Eastern Africa is part of sub‐Saharan Africa and conventionally divided into the Horn of Africa (Somalia, Djibouti, Eritrea, and Ethiopia) and East Africa (Kenya, Tanzania, and Uganda; Figure 1, see Methodology section). The region is home to over 160 different ethnic groups, each contributing unique fermented products. Many of these products are shared across countries but are known by different names, reflecting the region's cultural and agricultural diversity (Marcus et al., 2023). These products, including dairy‐based staples like *Ergo* and *Ititu* and plant‐based items like *Injera* and *Shameta*, are produced through spontaneous fermentation. This process relies on naturally occurring microorganisms, particularly lactic acid bacteria (LAB) and yeasts, to enhance the shelf life, safety, and nutritional value of the foods and eventually food security (Anyogu et al., 2021; Mulaw & Tesfaye, 2017; Figure 2). Fermentation can be exploited as a tool for increasing food security (Misci et al., 2021). In fact, it serves not only as a preservation method but also as a means to enrich food with bioactive compounds, probiotics, and essential nutrients, such as vitamins and minerals. LAB, for example, are known to synthesize vitamins like folate and riboflavin, reduce anti‐nutritional factors, and improve the bioavailability of iron and zinc, making fermented foods a key contributor to nutritional security in the region (Muyonga et al., 2018; Uusiku et al., 2010). The practice also aligns with sustainable development goals by supporting local economies, reducing food waste, and enhancing food accessibility. On the other hand, despite their widespread consumption and cultural importance, the production of traditional fermented foods in Eastern Africa remains largely artisanal, with non‐standardized practices leading to quality and safety challenges. These include contamination by foodborne pathogens (*E. coli*, *Salmonella*, *L. monocytogenes*) and the presence of mycotoxins in plant‐based products like fermented cereals. Recent reviews and studies have explored various aspects of fermented foods globally and regionally, providing valuable insights into their nutritional, microbial, and safety profiles. For instance, Anyogu et al. (2021) emphasize the need for standardization and the use of controlled starter cultures in African fermented foods to address issues of inconsistency and contamination. Another study by Tamang et al. (2020) offered a global perspective on traditional fermented foods, underscoring their socio‐economic importance and the need for deeper investigations into regional practices. Further studies focused on the microbiome of African fermented foods, revealing the presence of diverse microbial populations of fundamental, technological, and commercial interest that could be harnessed to further improve health, food safety, and quality (Ghosh et al., 2024; Obafemi et al., 2022). More focused research on Eastern African products is also available. For example, Misci et al. (2021) examined the metabolic pathways of LAB in traditional fermented foods, revealing mechanisms for enhancing food safety and quality. Comprehensive reviews, such as the work by Banwo et al. (2024), also addressed the dual challenges of food security and commercialization, identifying gaps in scaling traditional practices while preserving their authenticity.

Despite these advances, a critical review focusing on the unique fermentation practices, microbial dynamics, and safety challenges specific to Eastern Africa is lacking. While global reviews offer broad insights, they often fail to capture the nuances of Eastern Africa's agricultural diversity, socio‐economic context, and Indigenous knowledge systems. Moreover, few studies bridge the gap between traditional practices and modern food safety and commercialization standards.

This review aims to address these gaps by providing a comprehensive analysis of Eastern Africa's fermented foods and beverages. It critically evaluates their production technologies, nutritional contributions, and safety issues while exploring the potential for innovation and scale‐up. By integrating recent findings and highlighting areas for further research, this review seeks to advance understanding and guide efforts toward the sustainable development and global promotion of these culturally significant foods.

## 2 REVIEW METHODOLOGY

A systematic literature search was conducted using Google Scholar, PubMed, and Scopus. The inclusion criteria for the search were as follows: (a) scientific research articles or official reports focused on traditionally fermented foods and beverages from Eastern Africa, (b) the processing techniques used in their production, (c) the nutritional profiles of these products, and (d) safety‐related findings concerning these foods and beverages. The search keywords included “beverages,” “Eastern Africa,” “fermentation,” “milk,” “traditional food,” “food safety,” “nutritional value,” and “toxins.” Articles were screened for relevance based on title and abstract, regardless of their publication year. With few exceptions, most of the reviewed reports were published between 2000 and 2024.

Based on the available literature, the present work describes the most popular Eastern African traditional fermented foods and beverages. Tables 1 and 2 provide an overview of these common foods and beverages, along with the used raw materials.

**TABLE 1:** Raw materials and country of origin of Eastern African traditionally fermented foods.

Raw material	Local name of fermented food	Country of origin	Reference
Milk	*Ergo* ^a^	Ethiopia	Admasu et al. (2016)
Milk	*Ittitu* ^a^	Ethiopia	Hussien et al. (2021)
Milk	*Ayib* (*traditional cottage cheese*)^a^	Ethiopia	Ashenafi (2006)
Camel milk	*Dhanaan* ^a^	Ethiopia	Karssa et al. (2024)
Camel milk	*Suusac* ^a^	Kenya, Somalia Ethiopia	Farah et al. (2007); Berhe et al. (2017)
Milk	*Qibe* (*traditional butter*)^a^	Ethiopia	Gonfa et al. (2001)
Milk	*Neter Qibe* (*traditional ghee*)^a^	Ethiopia	Gonfa et al. (2001)
Milk	*Mursik* ^a^	Kenya	Nieminen et al. (2013)
Milk	*Amabere* ^a^	Kenya	Nyambane et al. (2014)
Milk	*Kule‐naoto* ^a^	Kenya	Mathara et al. (2004)
Milk	*Maziwa lala* ^a^	Kenya, Uganda, Tanzania	Bille et al. (2000)
Milk	*Kwerionik* ^a^	Uganda	Nakavuma et al. (2012)
Teff or sorghum	*Injera* ^b^	Ethiopia, Eritrea	Mohammed et al. (2011)
Enset/false banana	*Kotcho* ^b^	Ethiopia	Hunduma and Ashenafi (2011)
Barley grain	*T'ilho* ^b^	Ethiopia, Eritrea	Abraha et al. (2013)
Wheat	*Kitcha* ^b^	Eritrea	Tesfamariam and Hurlbert (2017)
Cornmeal or sorghum	*Muufo* ^b^	Somalia	Wolgamuth et al. (2022)
Mixture of flours cornmeal, sorghum	*Kimis* ^b^	Somalia	^*^PC
Mixture of sorghum, wheat or cornflour	*Canjeero* ^b^	Somalia	Wolgamuth et al. (2022)
Cassava	*Kivunde* ^b^	Tanzania	Kimaryo et al. (2000)
Sour milk and maize porridge	*Xwante*	Tanzania	Lorri and Svanberg (1995)
Fresh milk, maize, bananas	*Olshoro*	Tanzania	Lorri and Svanberg (1995)
Sorghum	*Magai* ^b^	Tanzania	Lorri and Svanberg (1995)
Cassava	*Odaga* ^b^	Tanzania	Lorri and Svanberg (1995)
Finger millet	*Obusera* ^b^	Tanzania	Lorri and Svanberg (1995)
Cassava	*Mokopa* ^b^	Uganda, Tanzania	Flibert et al. (2016)

^a^ Milk‐based fermented foods.

^b^ Plant‐based fermented foods.

* PC from personal communication with graduate students from their respective countries.

**TABLE 2:** Raw materials and country of origin of Eastern African traditionally fermented beverages and porridges.

Raw material	Local name of fermented beverage	Country of Origin	Reference
Barley (*Hordeum vulgare* ), “gesho” (*Rhamnus prinoides*), sorghum	*Areke*	Ethiopia, Eritrea	Debebe et al. (2017)
Malted and non‐malted barley (*H. vulgare*), “*gesho*” (*R. prinoides*)	*Korefe*	Ethiopia	Getnet and Berhanu (2016)
Barley	*Keribo*	Ethiopia	Abawari (2013)
Honey, “gesho” (*R. prinoides*)	*Tej*	Ethiopia	Tadesse et al. (2020)
Roasted barley (*H. vulgare*) flour, salt, linseed (*Linum usitatissimum* L.) flour, chili pepper (*Capsicum annuum*)	*Shameta*	Ethiopia	Akalu et al. (2017)
Barley (*H. vulgare* L.), wheat (*Triticum aestivum* L.), maize (*Zea mays* L.), finger millet (*Eleusine coracana* L.), sorghum (*Sorghum bicolor* L.), *“teff”* (*Eragrostis tef* L.), “gesho” (*R. prinoides*)	*Tella*	Ethiopia	Andualem et al. (2017)
Honey, bladder of cow	*Booka*	Ethiopia (Oromia Guji)	Elema et al. (2018)
Sorghum (*S. bicolor*), maize (*Z. mays*), finger millet (*E. coracana*), vegetables, root of taro (*Colocasia esculenta* L.)	*Cheka*	Ethiopia	Alemayehu (2018)
Sorghum, millet	*Suwa*	Ethiopia, Eritrea	Tadesse et al. (2020)
Porridge‐like dish from sorghum or corn	*Asseda*	Somalia	*PC
Azuki beans	*Cambuulo*	Somalia, Djibouti	Wolgamuth et al. (2022)
Maize, sorghum, wheat, finger millet, teff, and barley	*Borde*	Ethiopia	Abegaz et al. (2002)
Barley	*Garrobey*	Djibouti	*PC
Millet	*Marisa*	Djibouti	*PC
Finger millet/sorghum	*Togwa*	Tanzania	Mugula et al. (2003)
Sugar cane juice, “*Ikisha*” (*Kigelia* *africana*) and “*Kisapa*” (*Aloe pluridens*)	*Denge/dengclua*	Tanzania	Tarimo and Kaale (2023)
Cassava, germinating maize	*Gongo*	Tanzania	Francis et al. (2017)
Fermented porridge of germinated maize flour	*Kiambule*	Tanzania	Kubo (2014)
Fermented porridge of germinated finger millet flour	*Kimpumu*	Tanzania	Kubo (2014)
Germinated maize flour	*Komoni*	Tanzania	Tusekwa et al. (2000)
Fermented porridge of ripe banana	*Mbege*	Tanzania	Francis et al. (2017)
Fermented of ripe banana	*Orubisi/amarwa*	Tanzania	Tusekwa et al. (2000)
–	*Ulanzi*	Tanzania	Tusekwa et al. (2000)
–	*Waini*	Tanzania	Francis et al. (2017)
Sorghum	*Omuramba*	Uganda	Robert (2011)
Sorghum, honey	*Enturire*	Uganda	Lyumugabe et al. (2012)
Finger millet/sorghum	*Obutoko*	Uganda	Mukisa et al. (2012)
Finger millet/sorghum	*Obushera*	Uganda	Mukisa et al. (2010)
Finger millet	*Obuteire*	Uganda	Mukisa et al. (2012)
Green maize, millet, sorghum	*Kirario*	Kenya	Kunyanga et al. (2009)
Maize	*Mkarango*	Kenya	Mwizerwa et al. (2018)
Sorghum, millet	*Bushera*	Uganda	Muyanja et al. (2003)
Maize	*Bussa*	Kenya	Katongole (2008)
Millet	*Malwa*	Uganda	Muyanja et al. (2010)
Sorghum, maize	*Kwete*	Uganda	Muyanja et al. (2009)
Maize, millet, sorghum	*Uji*	Kenya	Wanjala et al. (2016)
Honey, “gesho” (*R. prinoides*)	*Mes*	Ethiopia, Eritrea	Tadesse et al. (2020)

*PC from personal communication with graduate students from their respective countries.

## 3 EASTERN AFRICAN TRADITIONAL FERMENTED FOODS

### 3.1 Traditional fermented milk‐based foods

Fermented milk and derived products traditionally produced in Eastern Africa are presented in Table 3, where the milk source, fermenting microbiota, and identification methods are reported. These traditionally fermented milks, produced by local farmers, provide essential nutrient requirements and income for milk producers and people in the region's countries (Moonga et al., 2022). These products are produced mainly through spontaneous fermentation at ambient temperatures (Akinyemi et al., 2021). The knowledge of microbiota involved in the fermentation of traditionally fermented products has increased in recent years, also thanks to advances in metagenomics, which have revolutionized our understanding of the microbial consortia driving traditional fermentations (Srinivas et al., 2022). Techniques such as 16S rRNA sequencing provide detailed insights into microbial diversity, enabling the identification of both dominant and subdominant species. Studies on African fermented products have revealed the presence of diverse LAB and yeast populations, including species that contribute unique functional properties, such as enhanced gut health and improved bioavailability of nutrients (Obafemi et al., 2022).

**TABLE 3:** Popular traditionally fermented milk and fermented milk products with associated microorganisms in Eastern Africa.

	Product local name						
Milk source	Fermented milk	Fermented milk product	Isolated microbiota	Identification methods	Country of origin	References	Main findings
Cow	*Amabere a* *maruranu*		*Lacticaseibacillus rhamnosus, S. thermophilus, L. bulgaricus, L. mesenteroides, L. plantarum, L. helveticus, Enterococcus faecium, S. cerevisiae, Rhodotorula mucoides, Candida famata*, and *C. albicans*	Phenotypic methods, 16S rRNA gene sequencing	Kenya	Boyiri (2014); Nyambane et al. (2014)	The product shows therapeutic and probiotic effects but has safety concerns
Cow	*Kule naoto*		*L. plantarum*, *L. fermentum, L. paracasei, L. acidophilus, L. mesenteroides, L. lactis*, and *E. faecium*	Phenotypic methods	Tanzania and Kenya	Mathara et al. (2004)	Variations in preparation methods affect product quality and safety
Cow	*Maziwa lala*		*Latilactobacillus curvatus, L. plantarum, Lactococcus cremoris, E. faecium, L. lactis, L. dextranicum*, and *L. mesenteroides*	Phenotypic methods	Kenya, Uganda, and Tanzania	Miyamoto et al. (2005)	Thirty‐nine lactic acid bacteria (LAB) strains were isolated from *Maziwa lala*
Cow	*Ergo*		*L. garvieae, L. lactis* subsp. *lactis, L. lactis, L. plantarum*, *L. fermentum*, and *Streptococcus* spp.	Phenotypic methods	Ethiopia	Assefa et al. (2008); Admasu et al. (2016)	Acidity increased due to the fermentation process, and chemical analysis revealed differences from Ethiopian standards
Cow	*Kwerionik*		*L. plantarum*, *L. paracasei* subsp. *paracasei*, *L. casei* subsp *casei*, *L. lactis* subsp. *lactis*, *L. mesenteroides* subsp. *mesenteroides*, and *E. faecium*	16S RNA gene sequencing	Uganda	Nakavuma et al. (2012)	Spontaneous fermentation involves the periodic removal of whey and the addition of fresh milk. LAB levels were initially high (10^9^ cfu/mL) but decreased over time to 10^5^ cfu/mL
Cow	*Ittitu*		*L. casei* and *L. plantarum*	Phenotypic methods	Ethiopia	Berhe et al. (2017)	LAB species play a critical role in fermentation, offering both preservation and probiotic benefits. The product is rich in nutrients and may support gut health benefits due to the presence of beneficial microbes
Camel	*Suusa (Suusac)*		*S. infantarius* subsp. *infantarius, E. faecium, L. helveticus, S. salivarius/thermophilus, Weissella confusa, L. fermentum, L. Lactis subsp. lactis, L. lactis, L. mesenteroides, L. curvatus, L. plantarum, L. salivarius, L. raffinolactis, L. mesenteroides* subsp. *mesenteroides, Wickerhamomyces famata, Pichia inconspicua, C. lusitaniae, Cryptococcus laurentii, R. mucilaginosa, S. cerevisiae, R. mucoides, Trichosporon cutaneum, C. krusei, R. penicillatum*	Rep‐PCR, 16s gene sequencing	Somalia, Kenya, and Ethiopia	Farah et al. (2007); Andualem and Geremew (2014)	It identifies the major LAB involved in camel milk fermentation and highlights the potential for growth in camel milk production through improvements in breeding, processing, and market access
Cow		*Ayib* *(Cottage cheese)*	*L. fermentum, L. plantarum, Kluyveromyces lactis, K. bulgaricus*, and *Lodderomyces pseudotropicalis*	Phenotypic methods	Ethiopia	Ashenafi (2006)	Rich in protein and low in fat, it contains beneficial LAB and yeasts but has high microbial loads due to raw milk and handling. Heat treatment reduces contamination and improves safety
Cow		*Qibe* *(traditional butter)*	NA	NA	Ethiopia	Andualem and Geremew (2014)	A traditional butter with high fat content (81.2%) and low protein. The fresh form is also used for cosmetics or fed to infants
Cow		*Mashita and Samuli*	*L. rhamnosus*	NA	Uganda	Abbo (2021)	It idenId Identifies *L. rhamnosus* as the dominant LAB. While this microbiota shows probiotic and antimicrobial potential, its low acid production in milk limits its use as a starter culture
Cow or goat	*Mursik*		*L. kefiri, C. krusei, L. casei*, *L. paracasei*, and *L. rhamnosus, Saccharomyces* species (*S. fermentati*), and *Metschnikowia sphaerica, L. fermentati*	16S rRNA sequencing	Kenya	Nieminen et al. (2013)	Fermentation produces significant levels of acetaldehyde and ethanol, with *C. krusei* and *L. kefiri* linked to higher acetaldehyde levels, potentially contributing to the high esophageal cancer incidence in Western Kenya

Abbreviation: NA, not available.

The range of African fermented foods being incredibly vast, the knowledge of the microbiology of those productions is sometimes limited. In the following section, fermented traditional food and beverages of Eastern Africa are discussed, with a focus on the fermenting microbiota. The same or comparable production methods are used across the region's countries, yet the final products may be known by different names.

#### 3.1.1 Amabere amaruranu

It is a traditional fermented bovine milk from Kenya, produced by the Kisii community. The migration of the Kisii community to urban areas in Kenya has spread the traditional production of *Amabere amaruranu* beyond its original region, demonstrating how traditional foods and practices can disseminate through migration, trade, and cultural exchange (Greiner & Sakdapolrak, 2013). The milk to be fermented is often boiled, cooled, and then added to the storage gourd (*Ekerandi)* for fermentation (Nyambane et al., 2014). However, recently, different containers, including plastics, have emerged for the fermentation of milk. Unlike some other products, this product involves the use of boiling milk before fermentation. The fermentation process is spontaneous; however, backslopping is commonly employed, in which a small batch of previously fermented milk (*Enduranerio*) is added to fresh milk to initiate the fermentation. Despite the fact that there is limited research available, yeasts and LAB such as *Lactobacillus* (45%), S*treptococcus* (25%), and *Leuconostoc* (20%) have been identified from *Amabere amaruranu* (Nyambane et al., 2014). Boyiri (2014) used 16S rRNA gene sequencing to identify a *Lacticaseibacillus rhamnosus* strain in *Amabere amaruranu* that exhibited potentially probiotic (bile‐stable, non‐mucinolytic, and antibacterial) activity. While there is no specific information documenting the mutualistic relationship between yeasts and LAB in *Amabere amaruranu*, Penna et al. (2015) reported that the acidic environment created by LAB promotes the proliferation of yeasts in milk. On the other hand, the growth of LAB is enhanced by the presence of yeasts, which may supply essential growth factors, including vitamins and soluble nitrogen compounds. Kotala and Onyango (2015) found that the cell extract of *L. rhamnosus* from *Amabere amaruranu* decreases the expression of a number of transcription factors associated with adipogenesis. At high dose levels (100 µL/mL of *L. rhamnosus* extract containing 10^7^ cfu/mL), the cell extracts were found to down‐regulate peroxisome proliferator‐activated receptor‐*ɤ*, sterol regulatory element‐binding protein 1, and adipose triglyceride lipase. In addition, the antibacterial effect of *Amabere amaruranu* against *E. coli* was also demonstrated by (Mokua, 2004).

#### 3.1.2 Mursik

It is another spontaneously fermented bovine milk product originating from the Kalenjin community in Kenya. Like *Amabere amaruranu*, the traditional production of *Mursik* has been promoted outside its native area through migration and cultural exchange (Samoei, 2015). As described by Nduko et al. (2016), the production of *Mursik* starts with the preparation of the traditional fermentation storage gourds, called *Sotet*, which are obtained from the branches of palm trees. After boiling and cooling down the milk, it is poured into the *Sotet*, followed by tightly covering it with a lid. The milk is left to ferment in a cool and dry place for 3–5 days or longer, depending on sensory preferences. To ensure a smooth and uniform consistency of *Mursik*, the *Sotet* is stirred during the fermentation process. *Mursik* is distinct from *Amabere amaruranu* in that the fermentation process includes regular stirring of the gourd, a practice not commonly seen in other fermented milk products. *Mursik* is often used for special events, such as marriages and success in athletics. According to Muigei et al. (2013), *Mursik* is also consumed by breast‐feeding mothers since it is believed to improve the immune system against common diseases. LAB (*Lentilactobacillus kefiri*, *L. casei, L. paracasei, L. rhamnosus, Levilactobacillus brevis, Lactobacillus helveticus*, and *Limosilactobacillus pontis*) and yeasts (*Cyberlindnera sphaerica, Candida krusei, C. kefyr*, and *Saccharomyces fermentati*) were reported from *Mursik*, contributing to its flavor and health promoting properties (Nieminen et al., 2013).

#### 3.1.3 Kule‐naoto

It is a traditional lactic fermented milk product consumed by the Maasai community in Kenya and northern Tanzania. Based on the latest available report, about 2–3 L of the fermented product are consumed per person per day (Nduko et al., 2016). According to Mathara et al. (2004), the product is made from unpasteurized whole milk from the Zebu breed of cows using centuries‐old methods. The fermentation gourd is gently rubbed with a flaming Olea Africana tree stick locally known as *Enkidogoe*, or from other trees, allowing the charcoal to break within. Lactobacilli and lactococci species were more prevalent LAB (10^7^–10^9^ cfu/mL), while enterococci were less prevalent (10^3^–10^4^ cfu/mL) in *Kule‐naoto* fermentation (Nduko et al., 2016). According to Isono et al. (1994) and Mathara et al. (2004), *Weissella confusa* was the dominant (81.2%) LAB species from *Kule‐naoto* in northern Tanzania, and only 6% of isolated strains were identified as *Leuconostoc* species.

#### 3.1.4 Maziwa lala

It is a yogurt‐like fermented milk, primarily produced and consumed by tribes engaged in livestock farming in Kenya, Uganda, and Tanzania. The production process involves washing the gourd with hot water and rubbing it with the burnt end of chopped sticks, letting the charcoal break inside. This process is repeated until the gourd becomes hot. The chopped stick, which is from a particular tree known as Mutamayio, is used for thermal treatment and flavoring. After the charcoal residues are removed, raw or boiled milk is poured into the gourd and left to ferment for 1–5 days (Nakamura et al., 1999). The use of thermal treatment distinguishes *Maziwa lala* from *Kule‐naoto* and *Mursik. Maziwa lala* contains *Lactobacillus, Streptococcus*, and *Leuconostoc* genera (T. S. Gebre et al., 2024).

#### 3.1.5 Ergo (sour milk)

It is a popular Ethiopian fermented whole milk, produced through a spontaneous fermentation process. This semi‐solid product resembles set yoghurt and has a pleasant odor, aroma, and taste. Its texture and flavor may vary within or among households (Gonfa et al., 2001). In urban areas, fermentation is typically left uncontrolled, with raw milk fermenting for 3 to 5 days at ambient temperature or in a warmer place (Ashenafi, 2002). However, household preparation of *Ergo* usually involves a 1‐day incubation at ambient temperatures. In contrast, rural areas, particularly pastoralists, use well‐smoked containers and previous fermentation milk as inoculum (Assefa et al., 2008), differently from *Mursik* and *Amabere amaruranu*. The incubation temperature usually does not vary significantly, and the taste of the fermented product typically remains consistent (Admasu et al., 2016). Similar to other products, *Ergo* undergoes spontaneous fermentation, involving *Streptococcus, Lactobacillus, Lactococcus*, and *Leuconostoc* species, which promote sourness and preservation (Ashenafi, 2002; Assefa et al., 2008).

#### 3.1.6 Ititu

It is a concentrated sour fermented milk consumed by the Borana tribes and Kereyu areas of Ethiopia. This pastoral community prioritizes the preparation of *Ititu* specifically during the rainy season, when milk is available in abundance (Gonfa et al., 2001). *Ititu* is similar to *Ergo* except that it is left to ferment spontaneously at ambient temperature up to 14 days in a large traditional fermentation vessel named “*Gorfa*.” Additionally, the separation of the whey from the fermented milk is the main manufacturing characteristics of *Ititu* (Berhe et al., 2017). Briefly, after coagulating the whole milk, the whey is removed using a wooden pipette. Then, a portion of fresh whole milk is added, and this process continues until enough curd is accumulated, that is, in about 14 days. *Ititu* can be stored for 15–20 days and has good keeping quality for about two months at ambient temperature (Berhe et al., 2017). The most prevalent LAB isolated from *Ititu* are *L. casei* and *L. plantarum* (Gonfa et al., 2001). While *Ergo* and *Ititu* share a similar microbial composition dominated by *Lactobacillus, Streptococcus*, and *Leuconostoc* spp., *Ititu* differs significantly in its longer fermentation period (up to 14 days) and the practice of whey separation.

#### 3.1.7 Kwerionik

In Uganda, milk fermentation is mostly carried out by pastoral groups such as the Bahima in the western part of the country. It is primarily made from Zebu or Ankole cow milk and fermented in a similar spontaneous manner to other regional products. *Kwerionik* is consumed within 7 days and like *Sussa* (as reported in Section 3.1.8), its consumption is usually higher during the dry season. Several LAB species *(L. plantarum*, *Enterococcus faecalis*, *L. paracasei* subsp. *paracasei*, *L. casei* subsp. *casei*, *L. lactis* subsp. *lactis*, *E. faecium* and *Leu. mesenteroides* subsp. *mesenteroides)* were identified from *Kwerionik* (Nakavuma et al., 2012).

#### 3.1.8 Sussa or Suusac

It is a fermented camel milk consumed not only by pastoral communities living in border areas of Somalia, Kenya, and Ethiopia but also in the arid and semi‐arid parts of Eastern Africa. Milk from camels plays an important role in pastoral communities (Mattiello et al., 2018). The use of camel milk differentiates *Sussa* from other products that rely on bovine milk. Recent research is focused on camel milk because of its medical application to prevent or help in treating several ailments such as autoimmune diseases, juvenile diabetes and allergies (Swelum et al., 2021). The milk is traditionally consumed either fresh or fermented. To prepare *Sussa*, the milk is left at ambient temperature for spontaneous fermentation, often in a covered container, for 24–48 h until it becomes sour (Farah et al., 2007). Identified LAB species in *Sussa* include *Latilactobacillus curvatus, L. plantarum*, *Ligilactobacillus salivarius*, *Lactococcus raffinolactis*, and *L. mesenteroides* subsp. *mesenteroides*. Yeasts such as *C. krusei, Geotrichum penicillatum*, and *Rhodotorula mucilaginosa* have also been isolated from the product, which contribute to its distinct fermentation profile (Andualem & Geremew, 2014).

#### 3.1.9 Other fermented milk byproducts

The consumption and reuse of traditional fermented milk byproducts is uncommon in Eastern Africa, with the notable exception of Ethiopia. These products, including *Ayib* (cottage cheese), *Metata Ayib* (spiced *Ayib*), *Qibe* (traditional butter), and *Nitir Qibe* (spiced ghee), play an important role in Ethiopian culinary traditions and daily life (Ashenafi, 2006). While these products share a common foundation in fermented milk, they differ significantly in their preparation, texture, uses, and stability. *Ayib*, for instance, is a typical acid‐heat‐coagulated cottage cheese made from defatted sour milk obtained after the churning of fermented whole milk (*Ergo*). It has a crumbly texture and is typically consumed fresh. In contrast, *Qibe* is a smooth, fatty butter made from fermented milk *(Ergo*) without separating the fat (Karssa et al., 2024). Unlike *Ayib*, which is consumed fresh, *Qibe* can be spiced and further processed into *Nitir Qibe*, a spiced ghee. Fresh *Qibe* has a distinct diacetyl flavor, but prolonged storage at room temperature can lead to putridity and rancidity. *Nitir Qibe*, however, is more stable due to its higher fat content and the preservation effects of spices. In addition to its culinary uses, unprocessed *Qibe* is also used for hairdressing and skin cosmetics, particularly by women. A small amount of fresh *Qibe* is also traditionally fed to infants of weaning age. A study on the isolation of *Lactobacillus* species from *Metata Ayib* has been published (Adugna & Andualem, 2023).

In Uganda, a product similar to *Qibe* is known as *Samuli*, which is made by heat clarification of *Mashita*, a milk‐fat product produced by the Bahima tribe. *Mashita* is prepared by churning *Makamo*, a fermented milk created by adding backslopping from previous batches and churning it in a large gourd (Hazra & Lodh, 2023). This process differs from Ethiopian practices, where *Qibe* is churned but not subjected to the same extended fermentation. *Mashita* is initially churned and then washed three times to remove off‐flavors before being used fresh or clarified into *Samuli* through heating and stirring. The final product, *Samuli*, shares some similarities with *Nitir Qibe* in its fatty consistency and uses in cooking, but the preparation methods and flavor profiles are distinct. The clarification process results in a unique flavor and texture that depends on the quality of the raw *Mashita* (Wani et al., 2022).

In Tanzania, *Olshoro* is a milk‐based fermented food consumed primarily by the Waarusha and Wameru tribes in the Arusha region. Unlike *Ayib* or *Qibe*, *Olshoro* is not just a fermented milk product but a fermented food made from dehulled maize, beans, and plantain (Lorri & Svanberg, 1995). Fresh milk is added to this mixture, and it is then left to ferment in a clay pot or gourd for 12–24 h. The fermentation of *Olshoro* does not primarily involve milk fermentation as seen in Ethiopian or Ugandan products. Instead, it involves the fermentation of the entire food mixture, with milk added for flavor and nutritional value. After fermentation, it is consumed within a few days, contrasting with the more long‐lasting shelf lives of products like *Qibe* and *Samuli. Olshoro* is primarily intended for children, reflecting its role in Tanzanian dietary traditions (Lorri & Svanberg, 1995).

### 3.2 Plant‐based foods and beverages

Traditional fermented foods and beverages/porridges are essential components of diets for Eastern African communities. The foods are derived from the local staple foods, usually a cereal such as maize (*Z. mays*), millet (*Pennisetum typhoideum*), sorghum (*Sorghum vulgare*), and sometimes a non‐cereal such as cassava *(Manihot esculenta)*, potato *(Ipomea tuberosum)*, or plantain (*Musa paradisica*). In the region, the staple foods are commonly prepared as a thick porridge, which is frequently made and served both as meals and beverages. The consumption of Indigenous alcoholic beverages constitutes over 80% of the total food and beverage consumption in the region (Willis, 2002). Both food and beverage products serve as an important source of cash income for women, contributing to their economic empowerment. Examples include *Orubisi* (Tanzania), *Bushera* (fermented sorghum, Uganda), and *Mes* (fermented honey wine, Ethiopia; EAC, 2023; Shayo et al., 2000; Tadesse et al., 2020).

#### 3.2.1 Injera

It is a flatbread made from cereals such as teff, wheat, barley, sorghum, maize, or a combination of these grains (Ashenafi, 2002). It is a main staple food in Ethiopia and also commonly consumed in Eritrea due to their historical and cultural ties with Ethiopia (Eritrea declared its independence from Ethiopia in 1993; Woldemikael, 2009). *Injera* is characterized by having “eyes” (honeycomb‐like holes) on its top surface due to the production and escape of carbon dioxide during fermentation and baking (Figure 5a). The most sensorially acceptable *Injera* is rich in eyes, soft, and thin and possesses a distinct sour taste resulting from fermentation (T. Girma et al., 2013). It is widely served during national and religious holiday celebrations as well as marriage, birthday, and funeral ceremonies. Compared to other fermented foods, *Injera* has a distinct texture and preparation method with, a two‐stage fermentation process lasting 30–72 h, depending on factors like ambient temperature, the concentration of *Ersho*, the starter culture from previous fermentation, and the type of fermentation container used (T. Girma et al., 2013; Neela & Fanta, 2020). The fermentation of *Injera* involves several LAB, such as *L. buchneri*, *L. casei*, *L. brevis*, *L. plantarum*, *L. fermentum*, *Companilactibacillus crustorum*, and *P. pentosaceus* (Neela & Fanta, 2020).

#### 3.2.2 Kivunde

**FIGURE 3:**
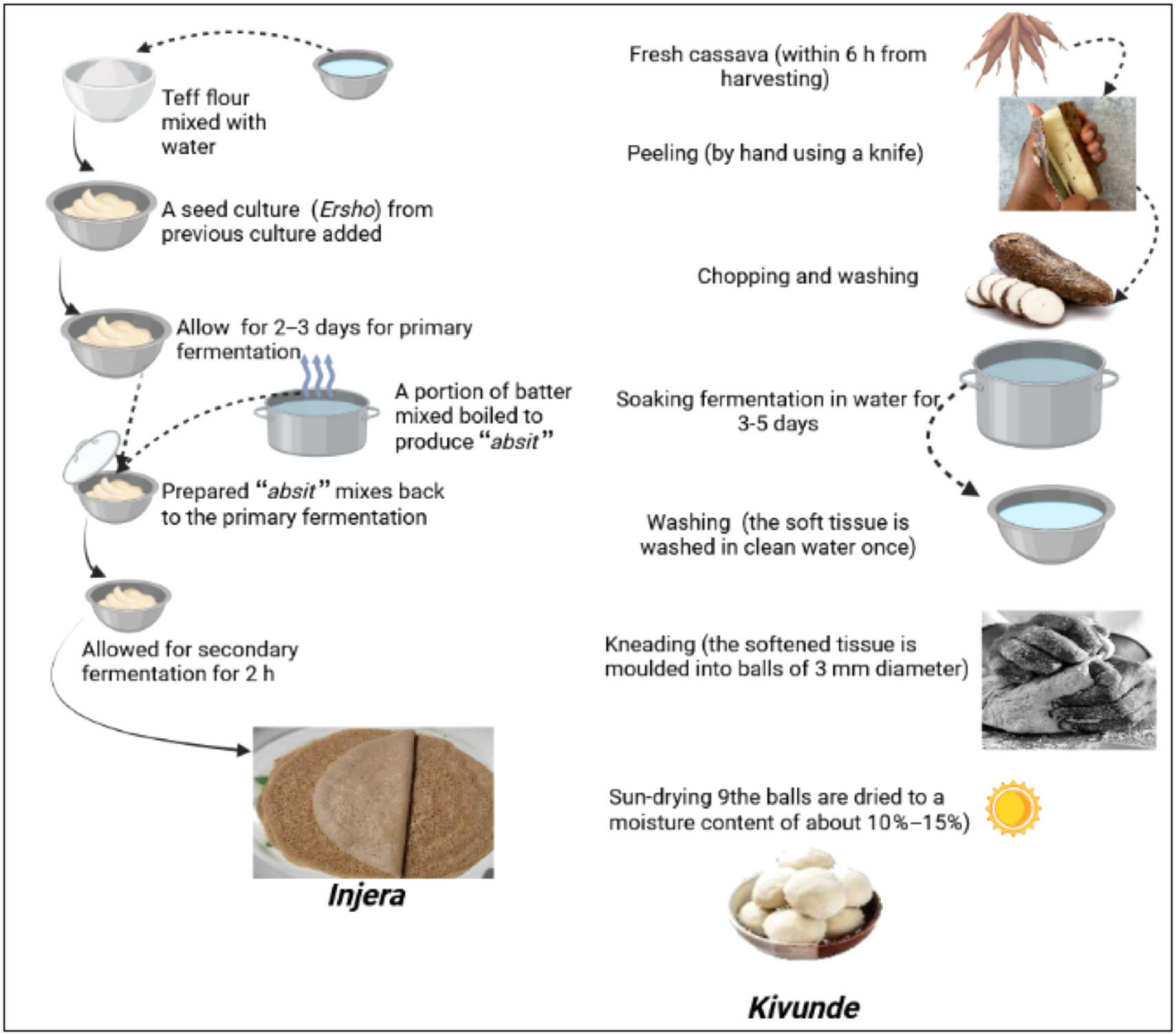
The traditional teff flour fermentation process for *Injera* preparation (Neela & Fanta, 2020) and *Kivunde* production (Kimaryo et al., 2000).

It is a popular traditional cassava fermented food produced in Tanzania. Figure 3 depicts the spontaneous and backslopping fermentation processes involved in *Kivunde* production (Kimaryo et al., 2000). To expedite fermentation, about 5% of leftover liquor from a previous *Kivunde* batch is added to the new mixture as backslopping to shorten the fermentation time. It has been reported that extending the fermentation period to 5 days results in a significant reduction in cyanide residual levels. In this regard, *Kivunde* shares similarities with other fermented starchy foods like *Mokopa* from Uganda, which also utilizes fermentation to improve nutritional content. Both products share a common goal of detoxifying cassava, yet they differ in their microbial profiles and fermentation techniques, with *L. plantarum* being the dominant microorganism in *Kivunde* fermentation, whereas *Mokopa* relies on the proteolytic contribution of molds (Oyewole, 2016).

#### 3.2.3 Kotcho

It is a traditional Ethiopian fermented food produced from parts of the “false banana” *Enset* (*Ensete ventricosum*), an herbaceous monocarpic plant. *Enset* has been cultivated for centuries in the region for food and fiber sources (Tiruha et al., 2014). The fermentation of *Kotcho* is a long‐term process, very different from the shorter fermentations seen in *Injera* and *Mokopa*. Scrapings from the leaf bases and pulverized stems and corms of *Enset* are combined and kneaded into a mash known as *Kotcho* in the region (Hunduma & Ashenafi, 2011). The mash is formed into a ball, wrapped in fresh *Enset* leaves, and stored at ambient temperature for 2–5 days. The mash is then deposited in a pit lined with fresh *Enset* leaves and fermented for a few weeks to several months or years depending on the incubation temperature. The formation of acids during *Kotcho* fermentation is attributed to LAB, *Enterobacteriaceae*, spore‐forming bacteria, and yeast (Tiruha et al., 2014).

#### 3.2.4 Mokopa

It is a traditional Ugandan cassava fermented food. As discussed by Flibert et al. (2016), *Mokopa* production starts from drying cassava roots for up to 2 hs after peeling and slicing them. Then they are covered with leaves and kept at ambient temperature to ferment for 3–4 days until the pieces become moldy. After the mold has been scraped off the fermented, moldy pieces are sun‐dried. Finally, the processed and dried pieces are milled into flour, which is subsequently made into *Kowan*, a food comparable to *Fufu* (a common food in western Africa). According to Oyewole (2016), the growth of mold on the root increases protein content in the final product by a factor of three to eight. While *Injera* and *Kivunde* use LAB as the dominant microbial agents in fermentation, *Mokopa's* fermentation is more aerobic, and its protein increase distinguishes it from the other products. On the other hand, no information is available on the microbial strains that are in charge of the fermentation. Other nations in East Africa, including Tanzania, Rwanda, and the Democratic Republic of the Congo, also widely use this aerobic fermentation technique (Kimaryo et al., 2000). The shelf life of *Mokopa* is extended by drying, which is not typical for fermented foods like *Borde* or *Shameta*, which have shorter shelf life due to their higher moisture content.

#### 3.2.5 Uji

It is the most popular non‐alcoholic porridge made and consumed by Kenyans. It is produced from unblended or composite flours of cassava and whole‐milled grains of maize, finger millet, or sorghum. *Uji* differs from *Injera* and *Mokopa* in that it is not a flatbread or solid food but a fermented liquid food, typically consumed as a beverage or a meal replacement. *Uji* is normally consumed as a breakfast meal or refreshment drink at any time of the day and is an important complementary food for children (Amadou et al., 2011). The preparation of fermented *Uji* starts by mixing unblended or composite flours with water to obtain a liquid slurry (30–40 g/100 Ml; Wanjala et al., 2016). The slurry is allowed to ferment spontaneously in a warm place (25–35°C) for 24–48 h. Fermentation is accelerated by using warm water (30–35°C) instead of cold water, adding sugar to the slurry, or inoculating the fresh slurry with previously fermented material (backslop culture). In contrast to *Uji, Togwa*, another fermented beverage from Tanzania, also uses malt flours and undergoes spontaneous fermentation, but *Togwa* is thicker and often consumed as a weaning food, while *Uji* is more common as a breakfast or snack food for children. Although lactobacilli are the predominant bacteria, yeasts also play a significant role in fermentation by supplying nutrients to the LAB, degrading raffinose and stachyose and adding desirable sensory properties (Jespersen, 2003).

#### 3.2.6 Enturire

**FIGURE 4:**
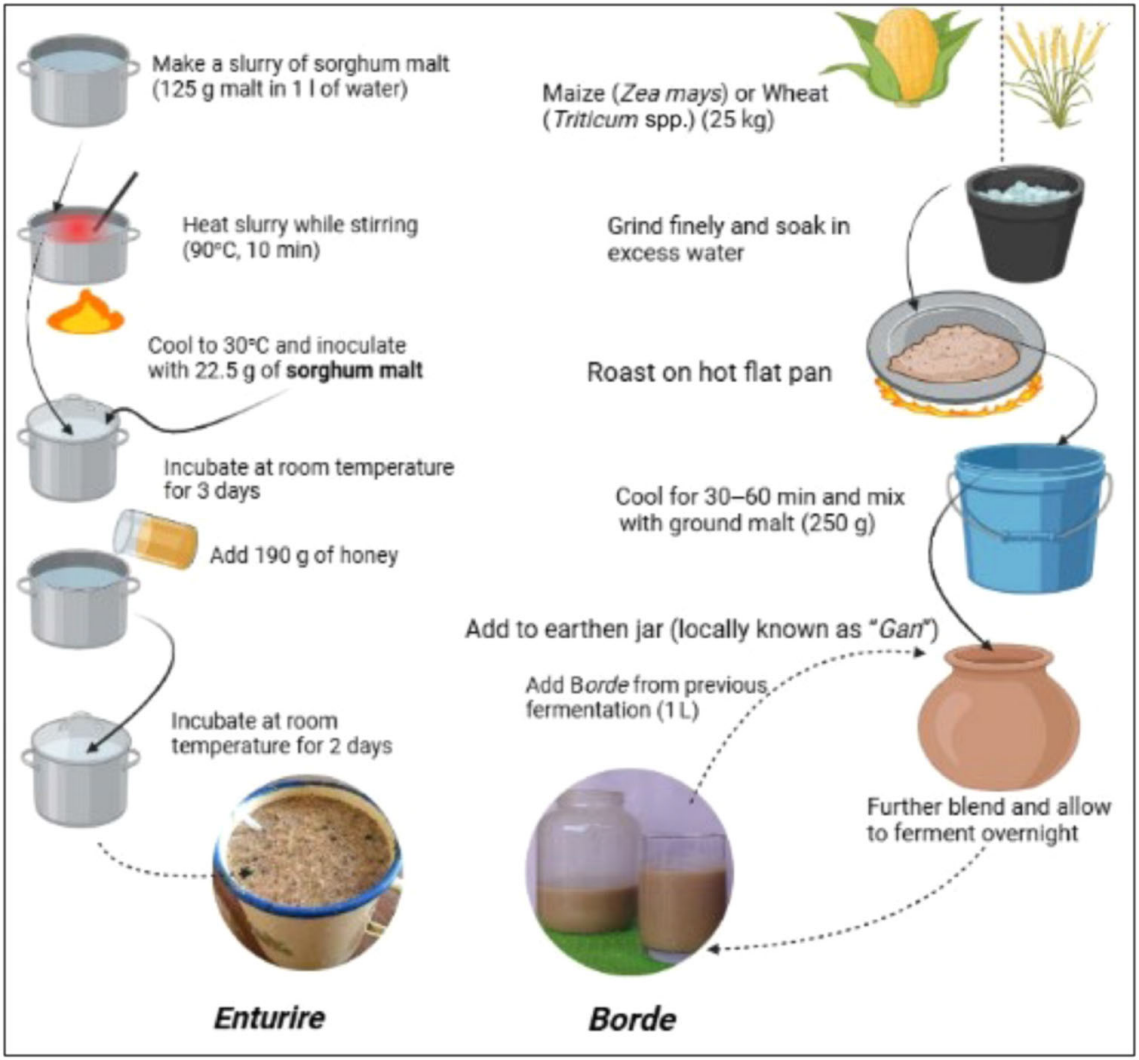
The traditional process used for spontaneous fermentation of alcoholic sorghum *Enturire* (Musika et al., 2010) and *Borde* (Nemo & Bacha, 2021).

It is a honey‐sorghum‐based sweet alcoholic beverage in Western Uganda. It is a member of the *Obushera* family of naturally fermented sorghum/millet beverages. Figure 4 illustrates *Enturire* fermentation (Mukisa et al., 2010). *Enturire* is processed by spontaneous fermentation, and like *Mes* from Eritrea and Ethiopia, it is an alcoholic drink prepared from locally available ingredients. The fermentation of *Enturire* allows for the growth of both useful and undesirable bacteria, making the quality and safety of the product variable (Lyumugabe et al., 2012). Similarly, *Mes*, made with honey and buckthorn, is also prone to microbial inconsistencies that can affect its final quality (Tadesse et al., 2020). However, *Mes* typically undergoes a longer fermentation period (up to 20 days), while *Enturire's* fermentation process can be as short as 4 days. *Mes* is primarily used for social occasions, much like *Enturire*, but the presence of honey in *Mes* gives it a sweeter and potentially more complex flavor profile than the more sorghum‐dominant flavor characterizing *Enturire*.

**FIGURE 5:**
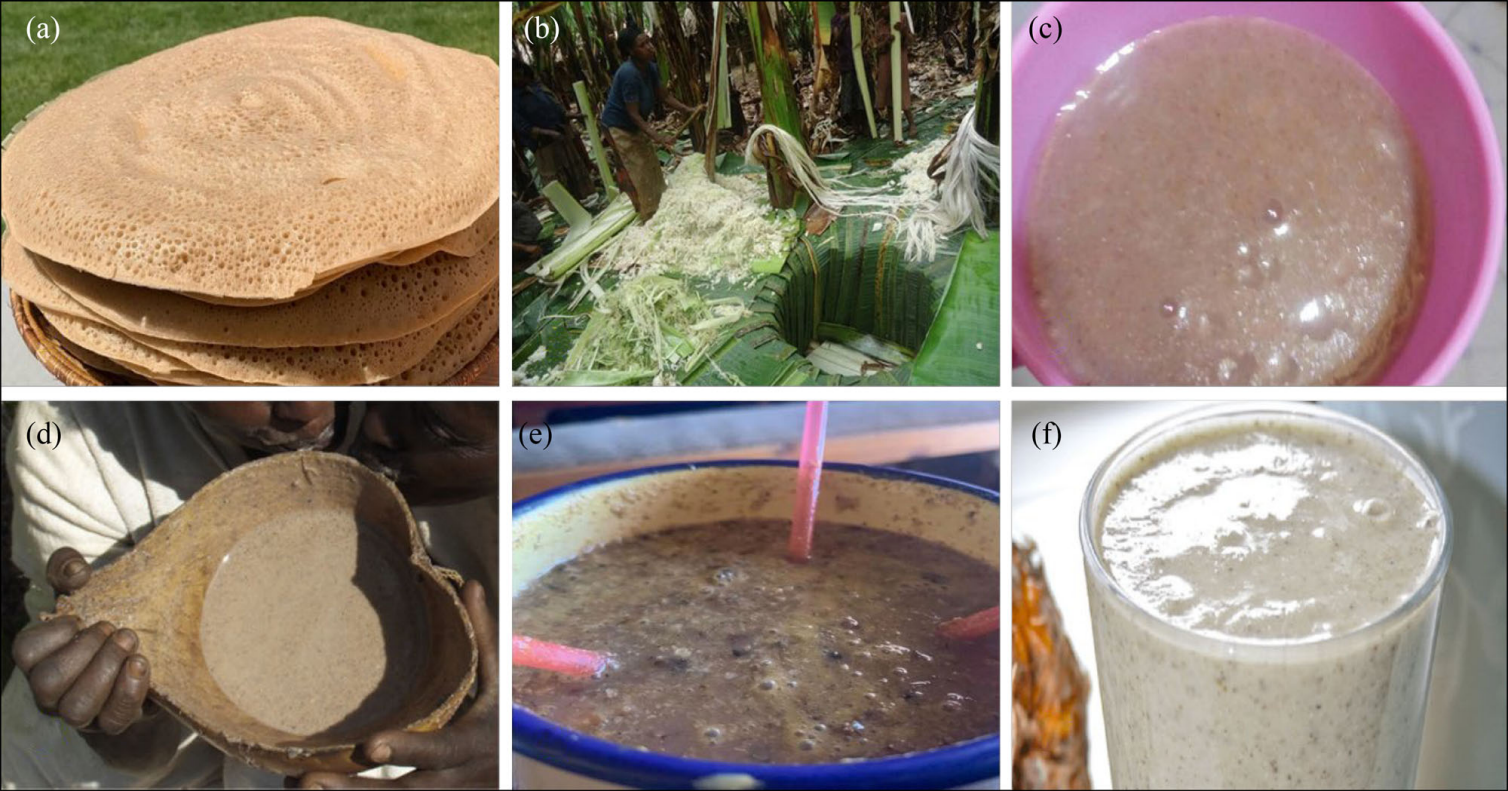
Some of traditionally fermented food and beverages in Eastern Africa. (a), *Injera* (*Source*: https://agameals.com/injera‐ethiopian‐sour‐flatbread‐recipe/); (b) decorticating pseudostem of *Enset* using a bamboo scrapperto make *Kotcho*, Angacha, Southern Ethiopia, (*Source*: Dalle & Daba, 2021); (c) Kenyan fermented porridge (*Uji*) (*Source*: https://michaelmorones.org/african‐food/1558‐tasty‐food‐kenyan‐uji‐porridge‐nigerian‐cuisine‐36.html); (d) *Borde* made of sorghum (*Source*: https://baskettoethiopia.wordpress.com); (e) *Enturire*, a traditional alcoholic beverage made from sorghum (*Source*: https://www.monitor.co.ug/uganda/magazines/life/enturire); (f) *Togwa* (*Source*: https://www.bluearrow.co.uk/blog/crazy‐fermented‐food).

#### 3.2.7 Borde

It is a traditional fermented low‐alcoholic beverage, mainly consumed in the southern and western parts of Ethiopia. It is made from various cereals like maize, barley, wheat, finger millet, sorghum, and teff by spontaneous fermentation using elementary equipment. It is similar to *Bushera* from Uganda, which is also made from sorghum and millet (Muyanja et al., 2003). However, *Borde* is typically thicker and has a sweet–sour taste, whereas *Bushera* can be adjusted to be sweeter or sour depending on the backslopping practices used (Muyanja et al., 2003). *Borde* is an opaque, whitish‐gray to brown‐colored beverage, with a thick consistency (Abegaz et al., 2002). Mothers are urged to consume large amounts of *Borde* after giving birth because local people think it improves breastfeeding (Debebe et al., 2017). According to Abegaz et al. (2002), *Borde* is mostly prepared from maize cereal, followed by wheat and barley. The production of *Borde* is summarized in Figure 4 (Nemo & Bacha, 2021). Abegaz et al. (2002) also reported *Lactobacillus* and *Streptococcus* species of LAB from *Borde* fermentation. Recent reports indicated that the addition of extracts of *Moringa oleifera* (drumstick tree) and *Thymus schimperi (Tosign)* to *Borde* enhanced the shelf life of the fermented product (Nemo & Bacha, 2021).

#### 3.2.8 Shameta

It is a low‐alcoholic (1.5%–02% v/v) homemade fermented porridge from Ethiopia, made from roasted barley and often used to support the strength and recovery of lactating women after childbirth. It is also consumed as a meal replacement, particularly for low‐income households. *Shameta* has been noted to have a short shelf life due to its high moisture content and is often too sour 4 h after it is ready for consumption (Kitessa et al., 2022). *Togwa*, another fermented beverage from Tanzania, is more stable and has a longer shelf life, largely due to the presence of *Lactobacillus* and *Leuconostoc* species, which help preserve the product (Mugula et al., 2003). Both products are rich in LAB, but *Shameta's* fermentation is significantly influenced by the use of spices such as Ethiopian caraway, false cardamom, and ground chilli, which also provide additional flavors and microbial diversity (Ashenafi, 2006). As described by Akalu et al. (2017), for *Shameta* production, about 110 kg of lightly roasted barley powder is mixed with ground linseed (9 kg), salt, and spices. Then, the mixture is allowed to ferment overnight. Ground linseed is responsible for the thick consistency of the final product. *Streptococcus, Pediococcus, Lactobacillus*, and yeasts are identified from *Shameta* fermentation (Table 4).

**TABLE 4:** Major plant‐based traditionally fermented foods and beverages produced in Eastern Africa.

	Fermented product local name						
Source	Food	Porridge/beverage	Isolated microflora and yeasts from fermentation	Alcoholic or non‐alcoholic	Country of origin	Reference	Main findings
Cassava	*Mokopa*		NA	–	Uganda	Flibert et al. (2016)	Fermentation increases the protein content of the final product by three to eight times
Pseudostem and corm of *Enset* (false banana)	*Kotcho*		*L. plantarum, Acetobacter* and *Levilactobacillus brevis*		Ethiopia	Weldemichael et al. (2019)	Kotcho samples varied in microbiota composition based on region and processing methods. The characteristic flavor and odor of *Kotcho* were attributed to short‐chain fatty acids produced by these microbes during fermentation
Tef/maize/sorghum	*Injera*		*Pediococcus pentosaceus, L. fermentum, L. piscium, L. plantarum, P. acidilactici, L. mesenteriodes* subsp. *mesenteriodes, L. raffinolactis, L. mesenteriodes* subsp. *dextranicum, E. cassiiflavus, S. cerevisiae, C. humilis, C. tropicalis, S. exiguus* and *P. norvegensis*	–	Ethiopia and Eritrea	Desiye and Abegaz (2013)	The fermentation batter for teff *Injera* involved both homo‐ and heterofermentative LAB and yeasts, which play a key role in defining its unique characteristics
Cassava	*Kivunde*		*L. plantarum*, *Lactococcus* species and yeasts	–	Tanzania	Kimaryo et al. (2000)	The study highlights the value of *L. plantarum* as a starter culture for improving the safety and quality of *Kivunde*
Finger millet		*Malwa*	*Lactobacillus* species	Non‐alcoholic	Uganda	Muyanja et al. (2010)	During fermentation, pH decreased, titratable acidity and ethanol increased, while carbohydrates and tannins decreased. Apparent increases in protein content suggest that fermentation enhances the nutritional value
Roasted barley, ground linseed, barley malt, and spices		*Shameta*	*Streptococcus, Lactobacillus, Pediococuus, Lactococcus, Micrococcus*, and *Leuconostoc, Saccharomyces* and *Rhodoturula* spp.	Low alcoholic	Ethiopia	Kittesa et al. (2023)	*Shameta*, consumed as a main dish or side dish, promotes breast milk production and recovery in lactating mothers. The combination of fermentation and cooking enhances its nutritional composition and physicochemical properties
Maize, sorgum, wheat, millet, tef, and barley		*Borde*	*W. confusa, L. brevis, W. viridescens, P. pentosaceus, P. pentosaceus* subsp. *intermidius (Saccharomyces)* and *Rhodoturula* spp.	Low alcoholic (3.35% v/v)	Ethiopia	Abegaz (2007)	The short shelf‐life of *Borde* and seasonal production fluctuations are major challenges for vendors. Production involves four key stages, with cereal type and malt proportions varying by locality based on availability, price, and consumer preferences
Maize, sorghum, and millet or cassava flours		*Uji*	*L. plantarum, Loigolactobacillus coryniformis*	Non‐alcoholic	Kenya/Uganda	Wanjala et al. (2016)	*Uji* is made from whole‐milled cereal flours to promote better health. Since most are prepared at home, product quality varies greatly
Germinated maize flour		*Togwa*	*L. brevis, L. fermentum, L.plantarum, P. pentosaceus, W. confusa, C. glabrata, C. pelliculosa, C. tropicalis, Issatchenkia orientalis, K. marxianus, P. anomala, S. cerevisiae*	Non‐alcoholic, sweet, burning taste	Tanzania	Mugula et al. (2003); Kubo (2014)	*Enterobacteriaceae* became undetectable within 24 h. Fructose decreased, while glucose increased during the first 12 h of fermentation. Organic acids detected included dl‐lactic, succinic, formic, pyruvic, citric, pyroglutamic, and uric acid
Fermented porridge of germinated maize flour		*Kiambule*	*Saccharomyces, Aspergillus, Candida, Escherichia*, and *Lactobacillus*	Slightly alcoholic (4% v/v) taste, sweet flavor	Tanzania	Francis et al. (2017); Kubo (2014)	The porridge types are mixed at the end to prevent bacterial contamination during fermentation. Germinated finger millet, with its high amylase activity, breaks down starch into fermentable sugars, facilitating fermentation
Germinated maize flour		*Komoni*	*Saccharomyces, Aspergillus, Candida, Penicillium, Escherichia, Lactobacillus*,	Alcoholic (5.27% v/v), sweet, burning taste	Tanzania	Francis et al. (2017); Kubo (2014)	Like *Kiambule*, the mixing technique prevents harmful bacterial contamination during fermentation
Fermented porridge of germinated finger millet flour		*Kimpumu*	*Saccharomyces, Aspergillus, Candida, Bacillus, Escherichia, Lactobacillus, Staphylococcus*, and *Streptococcus*	Slightly alcoholic (4% v/v) taste, sweet flavor	Tanzania	Kubo (2014)	Germinated finger millet, rich in amylase, is used for starch saccharification, converting starch into fermentable sugars
Fermented of ripe banana		*Orubisi/amarwa*	*Saccharomyces, Aspergillus, Candida, Penicillium, Bacillus, Escherichia, Lactobacillus, Staphylococcus*, and *Streptococcus*	Alcoholic (2.5% v/v) sweet and slightly hazy	North‐western Tanzania	Shayo et al. (2000)	The protein content of *Orubisi* increased from 2.0% to 2.7% after 120 h of fermentation. The beverage contained ethanol and iso‐butanol, but no methanol was detected
Maize		*Busaa*	*L. casei* var. *rhamnosus, L. helveticus, S. salivarius, P. damnosus, P. partulus, C. krusei*, and *S. cerevisiae*	Alcoholic (2‐4% v/v)	Kenya	Katongole (2008)	The beer contains 0.5%–1% lactic acid, contributing to its sour flavor. It is distinguished by its souring process, which occurs before malt addition, and its uncontrolled fermentation
Geen maize, millet, and sorgum		*Kirario*	*L. mesenteroides* ssp. *mesenteroides/detranicum, L. citreum, L. lactis* ssp. *lactis, L. raffinolactis, L. plantarum, L. brevis, Secudinlactobacillus collinoides*, and *W. cibaria*	Non alcoholic	Kenya	Kunyanga et al. (2009)	The initial pH of 6.4 dropped to 3.3 after 48 h of fermentation, indicating acidification from microbial activity. Both traditional and laboratory methods showed a high degree of hygiene with low or undetectable coliforms
Sorghum and millet		*Bushera*	*L. brevis, L. fermentum, L. plantarum, L. paracasei* subsp. *paracasei, L. delbrueckii* subsp. *delbrueckii, E. faecium, S. thermophilus*	Non alcoholic	Uganda	Muyanja et al. (2003)	Germinating grains before fermentation increases sugar concentration, protein content, and mineral levels in the final product. The variability in *Bushera* composition highlights the potential for standardizing production to consistency and quality
Fermented porridge of ripe banana		*Mbege*	*L. rhamnosus, L. helveticus, S. salivarius*, *Pediococcus, C. krusei*, and *S. cerevisiae*	Alcoholic (2.25 % v/v)	North Eastern Tanzania	Kubo and Kilasara (2016)	A traditional multi‐step brewing process where fermented banana porridge (*Nyalu*) provides yeasts and germinated finger millet porridge (*Mso*) supplies fermentable sugars
Maize and millet		*Kwete*	*L. plantarum, L. mesenteroides, S. cerevisiae, Schizosaccharomyces pombe*		Uganda	Muyanja and Namugumya (2009)	A significant decrease in pH, increase in titratable acidity, and a rise in ethanol content during fermentation contribute the characteristic taste and alcohol content of *Kwete*
Sorghum malt		*Enturire*	*S. cerevisiae, L. reuteri*	Alcoholic	Uganda	Mukisa et al. (2010); Mukisa et al. (2012)	*S. cerevisiae* was the most predominant
Millet, sorghum		*Obushera*	*Streptoccus gallolyticus, S. infantarius, L. fermentum, L. delbrueckii, W. confusa, L. reuteri, Clavispora lusitaniae, Cyberlindnera fabianii, I. orientalis, S. cerevisiae*	Non‐alcoholic	Uganda and Tanzania	Mukisa et al. (2012)	The fermentation process effectively reduced coliforms and increased LAB and yeast populations, enhancing both fermentation product safety

#### 3.2.9 Mes

It is a pleasant traditional fermented honey‐based beverage from Eritrea and Eastern Tigray region of Ethiopia, often prepared to mark special social events. Similar to *Tej*, another Ethiopian honey wine, *Mes* relies on honey as its primary raw ingredient. However, *Mes* distinguishes itself by incorporating buckthorn powder and *Tsedo* (tree bark), which are added to improve its flavor and shelf life. *Mes* undergoes a much longer fermentation period (up to 1 month), compared to the relatively shorter fermentation time of *Tej*. The variation in fermentation duration, along with the addition of specific local ingredients, contributes to a unique flavor profile that sets *Mes* apart from other honey‐based beverages. The fermentation barrel used for *Mes* production is usually smoked with *weyra* (*Olea europaea* subsp. *cuspidate*). *Mes* preparation starts with diluting 1 kg of honey with 2 L of water, followed by stirring and being left at room temperature for 4 days. After filtration of the wax, buckthorn powder and *Tsedo* (the bark of a tree) are added (Tadesse et al., 2020). The addition of *Tsedo* is to improve the quality and durability of *Mes*, which is often preferred by consumers. Sometimes, the fermentation period extends for 20 days at ambient temperature. In these situations, 1 kg of honey is added if the individual needs it prior to consumption. *Mes* is most like *Tej*. The major yeasts identified from *Tej* are *S. cerevisiae*, *K. bulgaricus*, *Debaromyces phaffi*, and *Kluyveromyces veronae*. The LAB consisted of *Lactobacillus, Streptococcus, Leuconostoc*, and *Pediococcus* species (Bahiru et al., 2006).

#### 3.2.10 Komoni (turbid beer), Kimpumu (straw beer), and Kiambule (hybrid straw beer)

They are Indigenous alcoholic beverages produced in Tanzania. These three beverages differ mainly in the grains used and the fermentation process. *Komoni* (turbid beer) and *Kiambule* (hybrid straw beer) are typically made from germinated maize (*kimea wa mahindi*) and finger millet (*kimea wa ulezi*). However, *Kimpuma* (straw beer) uses only germinated finger millet (Tarimo et al., 2023). The fundamental similarity between all three beverages is their use of germinated grains, which facilitate the fermentation process through the action of enzymes, although the specific microbial strains involved have yet to be identified. These beverages share a common goal of producing a mildly alcoholic, effervescent drink, often consumed as a local refreshment. The key difference lies in the type of grain used, which influences the texture and flavor, with maize‐based variants likely being more starchy and millet‐based variants potentially offering a more robust, grainy flavor. Details of the preparation methods of the three Indigenous alcoholic beverages are described in Supporting Information Figure S1 (Kubo, 2014).

#### 3.2.11 Orubisi/Amarwa

**FIGURE 6:**
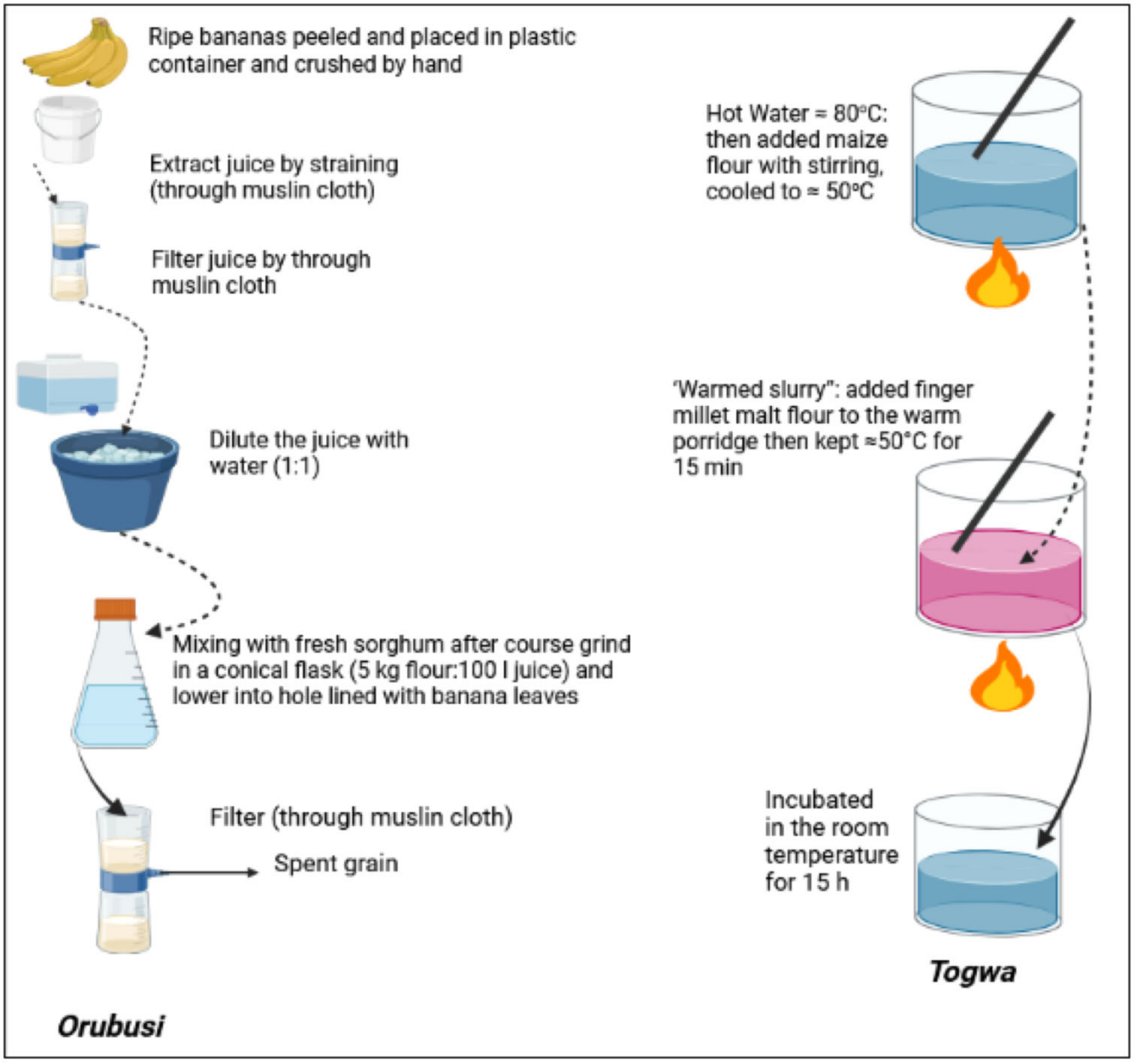
Traditional preparation of *Orubisi* (Shayo et al., 2000) and *Togwa* (Kitabatake et al., 2003) in the field of East Africa.

It is a traditional fermented beverage of the Haya (Wahaya) tribe in northwestern Tanzania. It is an effervescent, slightly sour alcoholic beverage made with bananas and sorghum. It is brown in color and has a consistency similar to very thin porridge. They are commonly chosen as a drink due to their contributions of various vital elements such as vitamin B group, minerals, proteins, and energy (Tusekwa et al., 2000). While *Orubisi* and *Mbege* (another Tanzanian alcoholic drink) both use locally available fruits and grains, the key difference lies in the main ingredients: *Orubusi* uses bananas, while *Mbege* uses finger millet. *Orubisi's* banana base gives it a sweeter, more fruity flavor, in contrast to *Mbege's* slightly more sour profile due to the millet's inherent tang. Additionally, the texture of *Orubisi* is more liquid and thin, similar to a very diluted porridge, whereas *Mbege* tends to be thicker. Figure 6 presents the preparation of *Orubisi* or *Amarwa* fermentation (Shayo et al., 2000).

#### 3.2.12 Togwa

It is a popular traditional fermented beverage in Tanzanian homes often used for weaning or as a refreshing drink. It is made from finger millet and maize flour (Figure 6), much like *Kirario*, a similar beverage from Kenya (Kitabatake et al., 2003). The significant difference between the two lies in the fermentation process: while *Togwa* is in a shorter time, *Kirario* involves the addition of sorghum or millet to green maize and is left to ferment for a slightly longer period (48 h). The product quality varies due to the spontaneous fermentation process (Mugula et al., 2001). Nowadays, lactic acid fermented porridge has attracted attention because of its microbial stability, improved nutritional value, and sensory properties and probiotic potential (Mugula et al., 2003). *L. plantarum, L. brevis, L. fermentum, P. pentosaceus, W. confusa, I. orientalis, S. cerevisiae, C. pelliculosa*, and *C. tropicalis* are isolated from *Togwa* fermentation (Mugula et al., 2003).

#### 3.2.13 Mbege

It is a traditional beer made from banana (*Musa* spp.) and finger millet (*E. coracana*). It is the most widely consumed traditional alcoholic beverage in northeastern Tanzania and plays a crucial role in the region's economy. Compared to *Orubisi*, which is also fermented from bananas and has a thinner, more porridge‐like consistency, *Mbege* is slightly thicker and more fermented, offering a stronger alcoholic kick (4% alcohol by volume after 48 h). Like *Orubisi, Mbege* undergoes fermentation with a combination of yeasts (*S. cerevisiae*) and LAB (*L. plantarum*), but *Mbege's* fermentation process and the presence of finger millet give it a more complex, slightly sour flavor. *Mbege* is consumed within 1 or 2 days. The ethanol concentration increased after 6 h of fermentation, and by 48 h, it reached 4.0%, while the Brix value dropped from 8.0 to 6.0 (Shayo et al., 1998). The detailed production steps are described in Figure S2.

#### 3.2.14 Kirario

It is an Indigenous, non‐alcoholic, lactic fermented porridge that is widely prepared and consumed in Kenya. The porridge is made from a blend of green maize with millet or sorghum, mixed in varying combinations and proportions. It shares similarities with *Togwa* (Tanzania) and *Bushera* (Uganda), which are also non‐alcoholic, lactic fermented beverages. The key difference lies in the choice of grains: *Kirario* is specifically made from green maize, whereas *Togwa* uses malted finger millet flour, and *Bushera* uses sorghum and millet. *Kirario* also stands out for its relatively low cost and widespread consumption in rural areas, making it a popular substitute for a meal. Additionally, *Kirario's* longer fermentation time (48 h) leads to a more pronounced tanginess, while *Togwa* may ferment quicker and be more variable in taste. Specifically, the production of *Kirario* included wet milling of green maize using a grinding stone followed by the addition of millet/sorghum and water. After further wet milling to finger particle sizes, the product is left for spontaneous fermentation at ambient temperature for 48 h. The product is one of the most popular cereal‐based traditional fermented beverages. Its consumption has expanded in the country since it serves as a low‐cost meal substitute, especially in rural communities (Kunyanga et al., 2009). The traditional *Kirario* product exhibited average counts of 9.63 Log cfu/mL for LAB, 8.62 Log cfu/mL for lactococci and 4.83 Log cfu/mL for yeasts and mold counts (Kunyanga et al., 2009).

#### 3.2.15 Bushera

It is the most commonly produced traditional non‐alcoholic beverage in Uganda's western highlands. It is produced at the village level from sorghum and millet by low‐income women for home consumption and sale. Like *Kirario*, it is a non‐alcoholic fermented beverage, but *Bushera*’s fermentation process is often accelerated through backslopping, which leads to a more acidic taste. This makes it distinct from *Kirario*, where fermentation is more controlled and typically results in a smoother, milder flavor. Typically, the Day‐1 fermented and completely fermented *Bushera* is usually consumed by young children and adults, respectively. This dual‐use aspect sets *Bushera* apart from *Kirario*, which is typically consumed by adults as a nutritious, low‐cost meal replacement. The production steps of sweet and sour sorghum *Bushera* are presented in Figure S3 (Muyanja et al., 2003). The backslopping practice in the production of *Bushera* is primarily employed in households that favor sour *Bushera* over sweet ones (Muyanja et al., 2003).

#### 3.2.16 Malwa (Ajon)

It is a non‐alcoholic beverage popular among the Iteso tribe in the eastern and northern parts of Uganda. Like *Bushera*, it is characterized by a short shelf life and a tendency to deteriorate quickly (Muyanja et al., 2010). Additionally, the shelf life and microbial stability of *Malwa* depend largely on the environmental conditions and the fermentation recipe, which makes it more variable in quality than other fermented beverages like *Togwa* or *Bushera*. The key difference between the two is the specific grains used: *Malwa* is primarily made from millet, whereas *Bushera* uses a combination of sorghum and millet. *Malwa* production is described in Figure S4 (Muyanja et al., 2010). During its fermentation, the prevailing conditions support the growth of numerous microorganisms, including LAB. LAB leads to the production of organic acids, which produce a sourdough. Souring is used to achieve basic biochemical changes (Abegaz et al., 2002).

## 4 NUTRITIONAL VALUE AND HEALTH

Despite the widespread consumption of traditional fermented foods and beverages in Eastern Africa, and the well‐documented potential role of these foods in child nutrition (Irakoze et al., 2021), there is limited information on their nutritional quality and composition. The nutritional composition of traditional fermented foods and beverages in Eastern Africa is influenced by both the raw materials and fermentation processes. These variations contribute to significant differences in the macronutrient and micronutrient profiles, as well as the bioavailability of nutrients, functional properties, and health benefits. Fermented food products can be grouped into five categories according to the main substrates or raw materials used in their processing: (1) Fermented animal proteins; (2) fermented starchy foods and cereals; (3) alcoholic beverages; (4) fermented legumes and oilseeds; (5) fermented vegetables (Achi, 2005; Hasan et al., 2014). Within the study region, it is notable that the first three categories are widely consumed.

Fermented animal products, such as *Ergo*, *Ititu*, *Sussac*, and *Mursik*, play a critical role in dietary protein intake, especially in pastoral and rural communities. Table 5 compares the composition of these products, highlighting differences in fat, protein, and lactose content, impacted by different fermentation processes. As an example, *Ititu* has a higher fat content (9.05%), compared to *Kule naoto* and *Mursik* due to additional milk added during fermentation (Berhe et al., 2017). Fermented milk dietary fats, in addition to providing concentrated energy, also serve as a major delivery vehicle for fat‐soluble vitamins and include a variety of fatty acids (e.g., conjugated linoleic acid) and bioactive components like triacylglycerols and phospholipids that are beneficial to health (Kris‐Etherton et al., 2010). Additionally, they are rich in minerals including Ca, Mg, P, and K, which may help in managing high blood pressure and cardio‐metabolic syndrome (Corgneau et al., 2017). The nutritional composition of fermented dairy products can also be affected by factors such as breed type, feed, and agro‐ecologies used in a given farming system (Ponnampalam et al., 2024). Camel milk‐based *Sussac* has a significantly higher protein content (7.17%) than cow fermented milk made in Kenya and Tanzania (Njage & Wangoh, 2008), which has been attributed to the naturally higher protein amount in camel milk (Hailu et al., 2016). In addition to giving pleasant taste and flavors to milk, fermentation is known as a cost‐effective method to enrich food with essential amino acids and vitamins that can help to prevent malnutrition (Holzapfel, 2002; Motarjemi, 2002). The fermentation process enhances the nutritional quality also by breaking down lactose, making these products suitable for individuals with lactose intolerance (Corgneau et al., 2017). Additionally, bioactive peptides, short‐chain fatty acids, and bacteriocins generated during fermentation improve gut health and regulate blood pressure (Chen et al., 2021; Mokua, 2004). Additionally, variations in fermentation processes and microbial activity could further enhance protein concentrations in camel milk. Recent research has focused on camel milk because of its medical application to prevent or help treating several ailments such as autoimmune diseases, juvenile diabetes, and allergies (Swelum et al., 2021).

**TABLE 5:** Chemical composition of some of traditionally fermented milk and fermented milk byproducts in Eastern African countries.

	Parameters							
Product name	pH	TA (%)	Fat (%)	Protein (%)	Lactose (%)	Ash (%)	Moisture (%)	Reference
*Ititu*	4.40	1.92	9.05	7.17	3.3–3.5	1.56	78.13	Berhe et al. (2017); Admasu et al. (2016)
*Kule naoto*	4.17–5.19	0.96	4.65	3.76	4.20^a^	0.94	ND	Mathara (1999); Nduko et al. (2016)
*Mursik*	3.46	1.51	5.42	6.39	3.95^a^	1.77	ND	Mathara (1999)
*Ayib*	3.3–4.6	ND	1.8	14.7	ND	0.9	80–81	Karssa et al. (2024)
*Metata Ayib*	4.0	0.43	28.7	43.7	ND	3.2	42.3	Seifu (2013)
*Arera*	ND	ND	1.4	3.1	3.4	0.6	91.5	EHNRI (1997)
*Dhanaan* ^a^	4.18	1.8	2.5	4.1	ND	1	ND	Kassa and Seifu (2012)
*Suusac*	3.6–4.4	ND	9.05	7.17	ND	0.74	ND	Njage and Wangoh (2008)
*Qibe*	ND	ND	81.2	1.3	0.1^a^	0.2	17.2	EHNRI (1997)

Abbreviations: ND, not determined; TA, titratable acidity.

^a^ Total carbohydrates.

Plant‐based fermented products, such as *Injera*, *Borde*, *Togwa*, and *Bushera*, are rich in carbohydrates and are an excellent source of energy. Additionally, these products can serve as probiotic carriers and hold potential as functional foods (Wuyts et al., 2020). Regarding the macronutrient composition of cereal‐based fermented products in the region, *Bushera* has the highest carbohydrate content, ranging from 77.7% to 85.7%, while *Togwa* has the lowest carbohydrate content at 12.5% (see Table 6). In terms of protein content, *Busaa* stands out with the highest protein level at 14.6%, while *Togwa* has the lowest protein content at 1.1%. The variations in these macronutrients levels can be attributed to differences in ingredients, fermentation processes, and regional practices (Yan et al., 2024).

**TABLE 6:** Chemical composition of some of traditionally fermented plant‐based foods and beverages in East African countries.

	Parameters							
Product name	Total protein (%)	Fat (%)	Carbohydrates (%)	Ash content (%)	Iron (mg/100 g)	Fiber (%)	Zinc (mg/100 gm)	Reference
*Injera*	12	2.40	24.99	1.57	9–24	1.95	2–43	Yegrem et al. (2021); Mohammed et al. (2011)
*Kotcho* ^a^	3.47	0.43	54.0	0.63	1.6	2.98	ND	Urga et al. (1997)
*Borde*	9.55	6.88	6.88	3.66	ND	ND	ND	Ashenafi and Mehari (1995)
*Shameta*	10.37	3.46	ND	6.85	8.1	ND	8.6	Ashenafi and Mehari (1995)
*Bushera*	9.63	1.8‐3	77.7–85.7	3.32	ND	4.3	ND	Muyanja et al. (2003)
*Malwa*	8.40	ND	70.36	ND	ND	ND	ND	Muyanja et al. (2010)
*Busaa*	14.6	ND	ND	ND	3.03	3.14	4.9	Adavachi (2017),
*Togwa*	1.1	0.2	12.5	0.2	ND	1.0	ND	Oi and Kitabatake (2003)
*Uji*	8.62	2.6	83.54	2.5	ND	2.9	2.17–6.9	Onyango (2014)
*Orubisi*	2.7	ND	ND	ND	ND	ND	ND	Shayo et al. (2000)
*Kivunde*	**–**	ND	ND	ND	ND	ND	ND	Kimaryo et al. (2000)

^a^ Seventh week of fermentation; ND, not determined.

Fermentation also enhances the bioavailability of minerals such as iron and zinc by reducing anti‐nutritional factors like phytates, tannins, and polyphenols. Most cereal‐based diets have poor nutrient bioavailability as a result of the presence of anti‐nutritional factors such as phytate/phytic acid (myoinositol‐1, 2, 3, 4, 5, 6‐hexakis dihydrogen phosphate), polyphenols, and tannins (Kumitch, 2019). Phytic acid impedes enzymatic activity such as trypsin and beta‐galactosidase and forms chelates with metal ions like Fe, Mg, Ca, and Zn, thereby reducing nutrient bioavailability (Samtiya et al., 2020). Similarly, tannins and polyphenols exert a strong negative impact on protein digestibility by forming complex protein carbonyl groups. Consequently, protein precipitation occurs and proteases are inhibited, leading to amino acid deprivation, particularly when the diet relies heavily on polyphenols‐rich cereal products (Nyanzi & Jooste, 2012). However, the Eastern Africa household‐level food and beverage production methods, such as decortication, soaking, germination, and lactic acid fermentation of cereals, can be used to reduce the amount of these inhibitors and thereby contribute to a more effective digestibility of plant proteins (Joye, 2019). Enzymes such as amylase, protease, lipase, and phytase play a crucial role in modifying the primary food products through the hydrolysis of polysaccharides, phytates, proteins, and lipids (Chelule et al., 2010). Phytases, which hydrolyze phytate into lower inositol phosphates, are present in most cereals and are believed to be activated during the germination and fermentation processes.

Low anti‐nutritional factors (polyphenols, phytate, and tannins) and high Ca (4.75 mg/100 g), Fe (3.95 mg/100 g), and Cu (0.7 mg/100 g) levels were reported by Nigatu and Gashe (1994) from plant‐based fermented food. In Kenya, Adinsi et al. (2017) showed that traditional fermentation increased mineral availability such as Fe, Mn, and Ca in both sorghum and finger millet. The process is also more effective in reducing phytic acid levels in sorghum and finger millet. Specifically, in sorghum grain, a mean decrease of 64.8% was observed after 96 h and 39.0% after 72 h. In finger millet, there was a mean reduction of 72.3% after 96 h and 54.3% after 72 h (Makokha et al., 2002). Cereal‐based fermented products are also a rich source of vitamins. While cereals naturally contain essential micronutrients such as specific vitamins, their fermentation with LAB or yeast strains can significantly increase their vitamin content (Kohajdová, 2017). For instance, Carrizo et al. (2016) isolated and assessed LAB strains from quinoa grains and quinoa sourdough for their production of B‐group vitamins. These microbial isolates exhibited significant phytase activity and produced riboflavin and folate. Similarly, high‐folate‐producing strains of *L. plantarum* have been found in *Injera* from Ethiopia (Tamene et al., 2019). Fermentation has significant potential to increase vitamin B12 in plant‐based foods, as certain microorganisms can produce it. However, further research is needed to explore the interactions between B12‐producing and non‐producing microbes and their impact on vitamin content. This could lead to optimized fermentation methods that enhance B12 levels and bioavailability. The fermentative microorganisms involved in cereal fermentation not only enhance sensory and nutritional qualities but also generate diverse metabolites that inhibit the growth of spoilage and pathogenic bacteria. These metabolites include organic acids like lactic acid, propionic acid, and acetic acid, which lower the initial pH value, thereby establishing within the food matrix. Consequently, this acidic environment contributes to prolonged shelf life of the fermented product (Nyanzi & Jooste, 2012).

Both alcoholic and non‐alcoholic fermented beverages are also widely consumed in the region and recognized by different names (Table 6). Fermentation enhances the nutritional content of these beverages, including both thick and thin porridges. The elevated levels of carbohydrate and protein in Ethiopian *Borde*, compared to other traditional beverages, support its reputation as a viable meal replacement for individuals unable to afford substantial meals for their daily needs (Hotessa et al., 2020). *Borde* stands out in terms of protein content, with 9.55%, which is higher than beverages like *Kotcho* (3.47%) and *Togwa* (6.8%). *Togwa*, however, has the lowest protein content among these products. In terms of fat content, *Borde* again leads with 6.88%, while *Shamita* contains 3.46%, and *Togwa* has the lowest fat content at just 0.2%. These differences highlight the potential of fermented beverages to provide varying levels of nutrients, including carbohydrates, proteins, and fats, depending on the product and its fermentation process. Korir et al. (1998) reported about 83.5% of soluble carbohydrate, 8.62% crude protein, 1.14 % crude fiber and 2.13% total ash from fermented Kenyan beverage *Uji* (thick porridge). *Togwa* is also rich in various trace elements such as Ca, Fe, K, and Na as well as high quantities of amino acids (Oi & Kitabatake, 2003). Enhanced levels of protein, carbohydrates, fats and essential minerals such as Fe, Mg, and Zn alongside reduced phenol and tannin concentrations were documented in *Bushera*, a traditional Ugandan fermented beverage. Fermented beverages also served as a significant source of energy, for instance, 56.1 kcal value attributed to *Togwa* beverages in Tanzania (Oi & Kitabatake, 2003).

The impact of African fermented foods on the nutrition, health and socio‐economy of the people of the continent was already suggested in the past (Franz et al., 2014), also linked to the possibility of developing probiotic products. African fermented foods represent a vast repository of potential health‐promoting microorganisms due to the diversity of substrates, fermentation methods, and microbial communities involved. Many LAB strains involved in Eastern African fermented products may exhibit antimicrobial efficacy against foodborne pathogens due to their bactericidal properties. Selected potential probiotic LAB strains (*L. plantarum* K132, *L. paracasei* K114, and *L. lactis* E124) from traditionally Ethiopian fermented foods were able to show a protective effect against *Salmonella* Typhimurium DT104 infection in experimental mice (Mulaw et al., 2020). Nigatu and Gashe (1994 also reported the antagonistic potential of fermented plant‐based foods against S*almonella* spp., *Pseudomonas aeruginosa*, and *Klebsiella* species, *Bacillus cereus* and *S. aureus*. The LAB belongs to the genera *Lactobacillus, Bifidobacterium, Enterococcus, Streptococcus*, and *Leuconostoc*, as well as strains like *L. plantarum* strain CIP 103151, *L. paracasei* strain NBRC 15889, and *L. plantarum* strain JCM 1149 inherently present in fermented *Borde* and *Shamita*, exhibited antimicrobial properties against various foodborne pathogens invading the gastrointestinal tract (Mulaw et al., 2019). Potential probiotic strains from fermented food have also been indicated to support the body's immune system, modulation of allergic diseases and treatment of infections formed during pregnancy (Bernardeau et al., 2008; Giraffa et al., 2010). Pinto et al. (2006) and Nielsen et al. (2010) identified *L. johnsonii* (BFE 6128, BFE 6154), *L. plantarum* (BFE 5092, BFE 5759, BFE 5878), *L. acidophilus*, *L. paracasei*, *L. rhamnosus*, *L. fermentum* as potential probiotic strains from fermented milk *Kule‐naoto* (Kenya) and *Kwerionik* (Uganda). The growth of *S. aureus* and *E. coli* in the cultured milk was inhibited by *L. lactis* spp*. lactis* and *W. confusa*, which were isolated from Masai fermented milk in northern Tanzania (Isono et al., 1994). LAB strains identified in Ethiopian fermented dairy products (*Ergo*, *Metata Ayib*, and *Ayib*)—namely, *L. acidophilus*, *L. paracasei*, *L. plantarum*, *L. rhamnosus*, *L. mesenteroides*, *S. thermophilus*, and *L. lactis* exhibited antimicrobial properties against a range of pathogens including *E. enteritidis*, *L. monocytogenes*, *P. aeruginosa, E. coli*, *S. aureus*, and *B. cereus* (A. Girma & Aemiro, 2021). In addition, the strain *L. rhamnosus* GR‐1, a probiotic suggested for women's health, was isolated from fermented milk in Tanzania (Van Tienen et al., 2011). Although many of these microorganisms demonstrate promising attributes, such as pathogen inhibition, improved lactose tolerance, and immune modulation, further research is needed to substantiate these claims through clinical trials. This will enable the classification of such microbes as probiotics, unlocking opportunities for their use in health‐focused applications (Obafemi et al., 2022).

Moreover, fermented foods offer a practical approach to modulating the gut microbiome, enhancing its resilience and functionality (Valentino et al., 2024). By providing beneficial bacteria and bioactive compounds, these foods have the potential to improve gut health, prevent diseases, and support overall wellness (Marco et al., 2017; Vinderola et al., 2023). Despite these advantages, African fermented foods remain underexplored, presenting untapped opportunities for both scientific discovery and local economic empowerment. Encouraging research and commercialization in this sector can bridge the gap between traditional knowledge and modern health applications.

## 5 SAFETY ISSUES

Although fermented foods are generally regarded as safe due to the antimicrobial properties of LAB and other fermenting microbes, safety challenges persist due to the artisanal nature of production (Aworh, 2023). These challenges include contamination by pathogens, toxin production, and inconsistent fermentation conditions.

While the acidic environment created during fermentation typically inhibits the growth of many foodborne pathogens (Achi, 2005), certain pathogenic and spoilage microorganisms can still thrive in fermented foods. These include spore‐forming bacteria like *Bacillus* spp. and *Clostridium* spp., non‐spore‐forming bacteria such as *L. monocytogenes*, *Salmonella* spp., and *Shigella* spp., bacterial toxin producers like *Staphylococcus aureus*, yeasts such as *Candida* spp. and *S. cerevisiae*, molds like *Rhizopus* spp. and *Penicillium* spp. and toxigenic fungi such as *Aspergillus* spp. (Oyedeji et al., 2023). These pathogens can persist in fermented products, especially when fermentation is poorly controlled or pH levels do not drop sufficiently. Studies indicate that some acid‐tolerant bacterial strains possess adaptive mechanisms to counteract acidic stress. These mechanisms include sensing acid stress, activating signal transduction pathways, maintaining intracellular pH homeostasis by regulating proton flow, repairing biological macromolecules, and regulating metabolism (Guan & Liu, 2020; Xu et al., 2023). These adaptations enable the bacteria to survive and proliferate in the acidic environments of fermented foods.

Fermented products involving spontaneous fermentations share common risks related to microbial contamination and the need for proper control over fermentation conditions such as temperature, pH, and time. Despite these commonalities, the impact of these risks can differ depending on the specific fermentation practices and the raw materials involved. For example, in *Mursik*, the addition of charcoal introduces polycyclic aromatic hydrocarbons and acetaldehyde, raising concerns about long‐term health effects, particularly esophageal cancer (Nieminen et al., 2013; Patel et al., 2013). In contrast, *Ayib* and *Ergo* are more likely to harbor high microbial counts, including coliforms and staphylococci, particularly if hygiene and fermentation processes are not strictly controlled. Table 7 presents some foodborne microbial pathogens and toxins reported in traditionally African fermented foods and beverages. Mamo et al. (2016) isolated a significant number of total aerobic mesophilic bacteria (9.1 Log cfu/mL), total spore count (8.6 Log cfu/mL), coliforms (4.5 Log cfu/mL), *Staphylococci* (4.7 Log cfu/mL), yeasts and molds (7.9 Log cfu/mL) from *Ergo* (Ethiopia). They also indicated homemade *Ayib* to contain a range of 2.4–4.8 log cfu/g of staphylococci. This highlights the variability in microbial risks among different African fermented foods. Although the backslopping process has been practiced in the production of various Kenyan fermented milk products, such as *Amabere amaruranu, Mursik*, *Kule naoto*, and *Suusa*, to maintain product consistency, the safety of these products remains unassured (Nduko et al., 2016). In Somalia, camel milk is consumed mainly in fermented form, and its spontaneous fermentation can result in undesirable products, some of which may be risky or dangerous for human health (Farah et al., 2007). Muyanja et al. (2009, 2010) detected coliforms during the early‐stage fermentation of *Kwete* and *Malwa* beverages in Uganda. Furthermore, improper handling and the use of unclean packaging materials may also introduce microbial hazards throughout the post‐processing stages (Adinsi et al., 2017).

**TABLE 7:** Indicator microorganisms and toxins isolated from Eastern African traditional fermented foods and beverages.

Fermented products	Country of origin	Microorganisms and toxins	Reference
**Milk products**			
*Ergo*	Ethiopia	Coliforms, yeast, and molds	Ashenafi, 2002
*Amabere amaruranu*	Kenya	Coliforms, *S. aureus*	Nyambane et al. (2014); Jans et al. (2017)
*Ayib* (cottage cheese)	Ethiopia	*B. cereus* and *S. aureus*	Ashenafi (2002)
**Plant‐based products**			
*Kotcho*	Ethiopia	*Micrococcus, Acinetobacter* spp.	Ashenafi and Abebe (1996); Birmeta et al. (2019)
*Shameta*	Ethiopia	*Staphylococcus* species	Kitessa et al. (2022)
*Obusera*	Uganda	*Escherichia coli, Staphylococci*, aflatoxins, coliforms	Byakika et al. (2019); Okoit (2022)
*Malwa/Ajon*	Uganda	Aflatoxins, coliforms	Okoit (2022)
*Makopa*	Uganda	*Aflatoxins*, fumonisins, zearalenone*, E. coli*	Abass et al. (2019); Lorri and Svanberg (1995) Abass et al. (2019)
*Kivunde*	Tanzania	Aflatoxins, fumonisins, zearalenone*, E. coli*	
*Bussa*	Kenya	Aflatoxin, fumonisin, deoxynivalenol*, S. aureus*, coliforms	Lorri and Svanberg (1995)
*Orubisi/Amarwa*	Tanzania	Coliforms	Muyanja et al. (2010)
*Uji*	Kenya, Tanzania, Uganda	Coliforms	Masha et al. (1998)
*Borde*	Ethiopia	Coliforms, Staphylococci	Ashenafi and Mehari (1995)
*Bongo*	Uganda	*Staphylococcus* spp., coliforms, Enterobacteriaceae	Mukisa et al. (2020)

Fermented beverages such as *Ulanzi, Togwa*, and *Denge* do not involve boiling, which raises the risk of contamination by unwanted microorganisms (Tusekwa et al., 2000). Additionally, studies have shown that hygienic practices such as hand hygiene, cleaning routines, waste management, keeping hair short or covering of hair, and wearing clean clothes are often not implemented, leading to contamination risks. This lack of attention to hygiene underscores a critical difference in safety management across different fermented foods. High microbial counts were encountered in the Tanzanian traditional alcoholic beverage *Orubisi*, although mainly represented by LAB (Shayo et al., 2000). In brief, viable counts included yeasts (7.3 Log cfu/mL), molds (6.87 Log cfu/mL), coliforms (2.07 Log cfu/mL), LAB (7.81 Log cfu/mL), and total aerobic count (7.46 Log cfu/mL).

Another safety concern for fermented African products is the potential presence of mycotoxins. While fermentation has been shown to reduce mycotoxin levels (Adavachi, 2017), their contamination has been documented in certain traditional foods and beverages (Table 7). Products relying on cereals, such as millet, sorghum, and maize, are particularly susceptible to aflatoxin contamination. This could be attributed to their rich nutrient composition and relatively high humidity that favors fungal growth (Pratiwi et al., 2015), as well as improper gain storage conditions. Aflatoxin, primarily produced by *Aspergillus flavus* and *A. parasiticus*, is the most commonly reported toxin in the region (Owaga et al., 2011). This issue holds significant public health importance due to its adverse effects on human health. Studies have found aflatoxin levels as high as 46 mg/kg in maize from Kenya and 19 mg/kg in Uganda (Anthony et al., 2012). Ayalew (2010) reported a mean total aflatoxin concentration of 5–27 µg/kg in maize from Ethiopia. In Tanzania, a majority (88%) of flour samples (maize, sorghum, and millet) were contaminated with total aflatoxins, exhibiting a log mean value of 1.5 ± 0.68 µg/kg, which surpassed the country's reference limit of 10 µg/kg (Nakuwa et al., 2023). In traditional fermented cereals, aflatoxins have been reported in prevalence ranges of 3%–75% (Misihairabgwi et al., 2018). The European Union has set maximum levels (MLs) for major mycotoxins in food products at high risk of contamination. For aflatoxins, the MLs in cereals and cereal products intended for direct human consumption are 2 µg/kg for AFB1 and 4 µg/kg for total aflatoxins (European Union, 2006).

Finally, for some specific fermented foods, such as those based on cassava, the most harmful component linked with their ingestion is cyanide (Montagnac et al., 2009), which can cause acute poisoning in humans. Some cassava varieties contain more than 10 mg of cyanide equivalents per kg of dry matter, mainly in the leaves (Flibert et al., 2016). Peeling, grating, soaking, boiling/cooking, ensiling, drying, and controlled fermentation are all techniques for detoxifying cassava (Kyawt et al., 2014), suggesting the need for a better usage of those techniques to eliminate cyanide from cassava roots‐based foods. In sum, while fermentation offers protective benefits through the growth of beneficial microbes, safety concerns remain diverse and require attention to the specific fermentation practices and the raw materials used.

## 6 FROM HOUSEHOLD TO SCALE‐UP

While most of the products discussed in this review are produced at the household level, they can potentially serve as the focus of large‐scale initiatives aimed at combating hunger, improving nutrition, and enhancing livelihoods (Materia et al., 2021). Scaling up traditional fermented foods presents both challenges and opportunities. Transforming artisanal production methods into small‐ and medium‐scale enterprises could offer significant economic benefits to communities, especially marginalized groups (Marshall & Mejia, 2011). One of the primary challenges in this scaling process is achieving standardization. Developing protocols for microbial safety and product consistency is essential. For example, using standardized fermentation vessels and starter cultures can enhance the predictability of the process while preserving traditional flavors (Moonga et al., 2020). In addition, infrastructure and training are crucial considerations. Investment in processing facilities, refrigeration, and transportation logistics is needed to maintain product quality, particularly for perishable items like fermented dairy products (Patel et al., 2014). Economic studies have also highlighted that reducing post‐harvest losses through fermentation can significantly improve food security and increase rural incomes (Wafula et al., 2016). Furthermore, expanding market access for traditional foods, which are typically produced locally for home consumption, is a challenge due to the lack of an established value chain and formal market structures. However, by branding and certifying these products as nutritious, culturally significant, and with extended shelf life, consumer trust and demand could be strengthened (Materia et al., 2021).

## 7 CONCLUSION AND FUTURE PERSPECTIVES

Traditional fermented foods and beverages in Eastern Africa represent a crucial part of the region's dietary, cultural, and economic landscape. This review highlights their diverse range, including dairy‐ and plant‐based products, which are primarily produced through spontaneous fermentation. The microbial dynamics of LAB and yeasts play a pivotal role in enhancing the nutritional value, shelf life, and sensory qualities of these products. However, challenges such as non‐standardized production processes, inconsistent quality, and safety risks from pathogens and toxins persist, limiting their broader adoption and commercialization. One of the central themes of this paper is thus the need for standardization of traditional fermentation practices, particularly in the context of ensuring quality and safety. However, it is critical to recognize that the feasibility and application of such standardization differ significantly between home and commercial production settings. For home production, which dominates in rural areas, the use of starter cultures or inoculants could pose economic challenges due to the associated costs. Instead, strategies such as controlled backslopping or the use of locally available materials to enhance fermentation consistency may be more practical. In contrast, commercial production has greater potential to adopt advanced technologies such as standardized starter cultures and mechanized processes, thereby ensuring consistency and scalability of products. Furthermore, introducing affordable and accessible starter culture kits tailored to specific regional needs could bridge the gap between these two production scales. This would empower small‐scale producers to enhance product quality while maintaining economic viability.

Besides the standardization of fermentation processes, by establishing controlled fermentation practices to ensure consistent quality and safety, key findings from this review emphasize the urgent need also for: (i) enhanced food safety measures by addressing contamination risks through improved hygiene, training for producers, and the development of robust safety protocols; (ii) commercialization and scaling up by exploring the techno‐economic feasibility of bringing these traditional foods to larger markets, both locally and internationally.

Despite their potential, knowledge gaps remain, particularly in the areas of microbial population dynamics, metagenomics, vitamins, minerals, and the optimization of fermentation processes for industrial applications. Future research should focus on harnessing advanced biotechnological tools to isolate functional microbes, explore probiotic potentials, and develop value‐added products. Additionally, integrating traditional knowledge with modern food science can pave the way for sustainable and scalable solutions.

In conclusion, the rich heritage of Eastern African fermented foods and beverages offers immense opportunities for nutrition, health, and economic growth. Addressing their production and safety challenges through interdisciplinary research and innovation will enable these foods to make a greater contribution to regional food security and global markets.

## AUTHOR CONTRIBUTIONS

**Habtamu Hawaz**: Conceptualization; writing—original draft; methodology; investigation. **Benedetta Bottari**: Validation; visualization; writing—review & editing; supervision. **Francesca Scazzina**: Validation; visualization; writing—review and editing; supervision. **Eleonora Carini**: Investigation; funding acquisition; writing—review and editing; validation; supervision.

## CONFLICT OF INTEREST STATEMENT

The authors declare no conflicts of interest.

## Supporting information



Method for the production of the turbid beer, straw beer and hybrid straw beer. Source, (Kubo, 2014).

Flow chart of traditional *Mbege* production Kubo and Kilasara 2016).

Production of Sweet and Sour sorghum *Bushera* (Muyanja et aL., 2003).

Flow chart for the traditional preparation of *Malwa* (Muyanja et al., 2010).

## References

[crf370137-bib-0001] Abass, A. B. , Adegoke, G. O. , Awoyale, W. , Gaspar, A. , Mlingi, N. , Andrianavalona, V. , Randrianarivelo, R. , Sulyok, M. , Mneney, A. , & Ranaivoson, L. R. (2019). Enumeration of the microbiota and microbial metabolites in processed cassava products from Madagascar and Tanzania. Food Control, 99, 164–170. 10.1016/j.foodcont.2018.12.025

[crf370137-bib-0002] Abawari, R. A. (2013). Microbiology of Keribo fermentation: An Ethiopian traditional fermented beverage. Pakistan Journal of Biological Sciences, 16(20), 1113–1121. 10.3923/pjbs.2013.1113.1121 24506010

[crf370137-bib-0003] Abbo, I. (2021). Probiotic, starter culture, and antimicrobial properties of lactic acid bacteria isolated from Ugandan traditional ghee [Doctoral dissertation, Kyambogo University].

[crf370137-bib-0004] Abegaz, K. (2007). Isolation, characterization and identification of lactic acid bacteria involved in traditional fermentation of Borde, an Ethiopian cereal beverage. African Journal of Biotechnology, 6(12), 1469–1478.

[crf370137-bib-0005] Abegaz, K. , Beyene, F. , Langsrud, T. , & Narvhus, J. A. (2002). Indigenous processing methods and raw materials of *borde*, an Ethiopian traditional fermented beverage. Journal of Food Technology in Africa, 7(2), 59–64. 10.4314/jfta.v7i2.19246

[crf370137-bib-0006] Abraha, A. , Uhlen, A. K. , Abay, F. , Sahlstrøm, S. , & Bjørnstad, A. (2013). Roasted barley foods: Processing and varietal differences affecting Kolo and Tihni, traditional grain products in Northern Ethiopia. Cereal Foods World, 58(2), 71–79. 10.1094/CFW-58-2-0071

[crf370137-bib-0007] Achi, O. K. (2005). The potential for upgrading traditional fermented foods through biotechnology. African Journal of Biotechnology, 4(5), 375–380.

[crf370137-bib-0008] Adavachi, W. M. (2017). The role of fermented maize‐based products on nutrition status and morbidity of children 6–59 months old in Western Kenya [Doctoral dissertation, University of Nairobi].

[crf370137-bib-0009] Adinsi, L. , Mestres, C. , Akissoé, N. , Vieira‐Dalodé, G. , Anihouvi, V. , Durand, N. , & Hounhouigan, D. J. (2017). Comprehensive quality and potential hazards of gowe, a malted and fermented cereal beverage from West Africa: A diagnostic for a future re‐engineering. Food Control, 82, 18–25. 10.1016/j.foodcont.2017.06.019

[crf370137-bib-0010] Admasu, M. A. , Cione, E. , & Aquaro, S. (2016). Microbiological characteristics and physico‐chemical parameters of fermented milk product ergo—A traditional yogurt product of Ethiopia. Food Science and Quality Management, 49, 42–45. https://hdl.handle.net/20.500.11770/133851

[crf370137-bib-0011] Adugna, M. , & Andualem, B. (2023). Isolation, characterization and safety assessment of probiotic lactic acid bacteria from metata ayib (Traditional spiced cottage cheese). Food and Humanity, 1, 85–91. 10.1016/j.foohum.2023.05.002

[crf370137-bib-0013] Akalu, N. , Assefa, F. , & Dessalegn, A. (2017). In vitro evaluation of lactic acid bacteria isolated from traditional fermented Shamita and Kocho for their desirable characteristics as probiotics. African Journal of Biotechnology, 16(12), 594–606. https://doi/10.5897/AJB2016.15307

[crf370137-bib-0014] Akinyemi, M. O. , Ayeni, K. I. , Ogunremi, O. R. , Adeleke, R. A. , Oguntoyinbo, F. A. , Warth, B. , & Ezekiel, C. N. (2021). A review of microbes and chemical contaminants in dairy products in sub‐Saharan Africa. Comprehensive Reviews in Food Science and Food Safety, 20(2), 1188–1220. 33506591 10.1111/1541-4337.12712

[crf370137-bib-0015] Alemayehu, H. G. (2018). Physico‐chemical characterization of commercial local alcohol beverages available in south nations, nationalities and People's regional state, Ethiopia. International Journal of ChemTech Research, 11(8), 227–231.

[crf370137-bib-0016] Amadou, I. , Gbadamosi, O. S. , & Le, G. W. (2011). Millet‐based traditional processed foods and beverages—A review. Cereal Foods World, 56(3), 115.

[crf370137-bib-0017] Andualem, B. , & Geremew, T. (2014). Fermented Ethiopian dairy products and their common useful microorganisms: A review. World Journal of Agricultural Sciences, 10(3), 121–133.

[crf370137-bib-0018] Andualem, B. , Shiferaw, M. , & Berhane, N. (2017). Isolation and characterization of *Saccaromyces cervisiae* yeasts isolates from “tella” for beer production. Annual Research & Review in Biology, 15(5), 1–12. 10.9734/ARRB/2017/34129

[crf370137-bib-0019] Anthony, M. H. , Francis, D. M. , Berka, N. P. , Ayinla, G. T. , & Haruna, O. G. (2012). Aflatoxin contamination in foods and feeds: A special focus on Africa. Trends in Vital Food Control Engineering, 10, 24919. 10.5772/24919

[crf370137-bib-0020] Anyogu, A. , Olukorede, A. , Anumudu, C. , Onyeaka, H. , Areo, E. , Adewale, O. , & Nwaiwu, O. (2021). Microorganisms and food safety risks associated with indigenous fermented foods from Africa. Food Control, 129, 108227. 10.1016/j.foodcont.2021.108227

[crf370137-bib-0022] Ashenafi, M. (2002). The microbiology of Ethiopian foods and beverages: A review. SINET: Ethiopian Journal of Science, 25(1), 97–140. 10.4314/sinet.v25i1.18076

[crf370137-bib-0023] Ashenafi, M. (2006). A review on the microbiology of indigenous fermented foods and beverages of Ethiopia. Ethiopian Journal of Biological Sciences, 5(2), 189–245. 10.4314/ejbs.v5i2.39036

[crf370137-bib-0024] Ashenafi, M. , & Abebe, Y. (1996). Microbial load and incidence of *Staphylococcus aureus* in market Bulla and Kotcho, traditional Ethiopian processed food products from Enset. Ethiopian Journal of Health Development, 10(2), 117–122.

[crf370137-bib-0025] Ashenafi, M. , & Mehari, T. (1995). Some microbiological and nutritional properties of Borde and Shamita, traditional Ethiopian fermented beverages. Ethiopian Journal of Health Development, 9(2), 105–110.

[crf370137-bib-0026] Assefa, E. , Beyene, F. , & Santhanam, A. (2008). Isolation and characterization of inhibitory substance producing lactic acid bacteria from Ergo, Ethiopian traditional fermented milk. Livestock Research for Rural Development, 20(3), 1–9.

[crf370137-bib-0027] Aworh, O. C. (2023). African traditional foods and sustainable food security. Food Control, 145, 109393. 10.1016/j.foodcont.2022.109393

[crf370137-bib-0028] Ayalew, A. (2010). Mycotoxins and surface and internal fungi of maize from Ethiopia. African Journal of Food, Agriculture, Nutrition and Development, 10(9), 4109–4123.

[crf370137-bib-0029] Bahiru, B. , Mehari, T. , & Ashenafi, M. (2006). Yeast and lactic acid flora of *tej*, an indigenous Ethiopian honey wine: Variations within and between production units. Food Microbiology, 23(3), 277–282. 10.1016/j.fm.2005.05.007 16943014

[crf370137-bib-0030] Banwo, K. , Adebo, O. A. , & Falade, T. D. (2024). Examples of lactic‐fermented foods of the African continent. In G. Venderola , A. Ouwehand , S. Salminen , & A. von Wright (Eds.), Lactic acid bacteria (pp. 312–327). CRC Press.

[crf370137-bib-0032] Berhe, T. , Vogensen, F. K. , Ipsen, R. , Seifu, E. , Kurtu, M. Y. , & Hansen, E. B. (2017). Traditional fermented dairy products of Ethiopia: A review. East African Journal of Sciences, 11(2), 73–80.

[crf370137-bib-0033] Bernardeau, M. , Vernoux, J. P. , Henri‐Dubernet, S. , & Guéguen, M. (2008). Safety assessment of dairy microorganisms: The *Lactobacillus* genus. International Journal of Food Microbiology, 126(3), 278–285. 10.1016/j.ijfoodmicro.2007.08.015 17889388

[crf370137-bib-0034] Bille, P. G. , Vovor, M. N. , Goreseb, J. , & Keya, E. L. (2000). Evaluating the feasibility of adding value to goat's milk by producing yoghurt using a low‐cost technology method for rural Namibia. Journal of Food Technology in Africa, 5(4), 139–144. 10.4314/jfta.v5i4.19272

[crf370137-bib-0035] Birmeta, G. , Bakeeva, A. , & Passoth, V. (2019). Yeasts and bacteria associated with kocho, an Ethiopian fermented food produced from enset (*Ensete ventricosum*). Antonie Van Leeuwenhoek, 112, 651–659. 10.1007/s10482-018-1192-8 30368690 PMC6418067

[crf370137-bib-0036] Boyiri, B. B. (2014). *Probiotic potential of bacterial isolates from ‘amabere amaruranu’ cultured milk* (Paper No. 2389) [Master's thesis, East Tennessee State University]. Electronic Theses and Dissertations. https://dc.etsu.edu/etd/2389

[crf370137-bib-0037] Byakika, S. , Mukisa, I. M. , Byaruhanga, Y. B. , Male, D. , & Muyanja, C. (2019). Influence of food safety knowledge, attitudes, and practices of processors on microbiological quality of commercially produced traditional fermented cereal beverages: A case of *Obushera* in Kampala. Food Control, 100, 212–219. 10.1016/j.foodcont.2019.01.024

[crf370137-bib-0038] Carrizo, S. L. , de Oca, C. E. M. , Laiño, J. E. , Suarez, N. E. , Vignolo, G. , LeBlanc, J. G. , & Rollán, G. (2016). Ancestral Andean grain quinoa as a source of lactic acid bacteria capable of degrading phytate and producing B‐group vitamins. Food Research International, 89, 488–494. 10.1016/j.foodres.2016.08.013 28460943

[crf370137-bib-0039] Chelule, P. K. , Mbongwa, H. P. , Carries, S. , & Gqaleni, N. (2010). Lactic acid fermentation improves the quality of amahewu, a traditional South African maize‐based porridge. Food Chemistry, 122(3), 656–661. 10.1016/j.foodchem.2010.03.026

[crf370137-bib-0040] Chen, L. , Wang, L. , Li, J. , & Shu, G. (2021). Antihypertensive potential of fermented milk: The contribution of lactic acid bacteria proteolysis system and the resultant angiotensin‐converting enzyme inhibitory peptide. Food & Function, 12(22), 11121–11131. 10.1039/d1fo02435c 34657947

[crf370137-bib-0042] Corgneau, M. , Scher, J. , Ritie‐Pertusa, L. , Le, D. T. , Petit, J. , Nikolova, Y. , & Gaiani, C. (2017). Recent advances on lactose intolerance: Tolerance thresholds and currently available answers. Critical Reviews in Food Science and Nutrition, 57(15), 3344–3356. 26713460 10.1080/10408398.2015.1123671

[crf370137-bib-0043] Dalle, G. , & Daba, D. (2021). Diversity and uses of enset (*Ensete ventricosum* (Welw.) Cheesman) varieties in Angacha district, Southern Ethiopia: Call for taxonomic identifications and conservation. Genetic Resources and Crop Evolution, 68, 485–498. 10.1007/s10722-020-00998-1

[crf370137-bib-0044] Debebe, A. , Chandravanshi, B. S. , & Abshiro, M. R. (2017). Assessment of essential and non‐essential metals in Ethiopian traditional fermented alcoholic beverages. Bulletin of the Chemical Society of Ethiopia, 31(1), 17–30. 10.4314/bcse.v31i1.2

[crf370137-bib-0045] Desiye, A. , & Abegaz, K. (2013). Isolation, characterization, and identification of lactic acid bacteria and yeast involved in fermentation of teff (*Eragrostis tef*) batter. Advanced Research in Biological Sciences, 3, 36–44.

[crf370137-bib-0047] East African Community (EAC) . (2023). Agriculture and food security . https://www.eac.int/agriculture

[crf370137-bib-0048] EHNRI, F. (1997). Composition table for use in Ethiopia (Part III and IV) . https://library.au.int/food‐composition‐table‐use‐ethiopia‐part‐iii‐5

[crf370137-bib-0049] Elema, T. , Olana, B. N. , Elema, A. B. , & Gemeda, H. F. (2018). Indigenous processing methods, physical properties, and proximate analysis of fermented beverage of honey wine booka in Gujii, Ethiopia. Journal of Nutritional Food Science, 8(669), 2. 10.4172/2155-9600.1000669

[crf370137-bib-0050] European Union . (2006). Commission Regulation (EC) No 1881/2006 of 19 December 2006 setting maximum levels for certain contaminants in foodstuffs (text with EEA relevance). Official Journal of the European Union, 24(364), 365–324. http://extwprlegs1.fao.org/docs/pdf/eur68134.pdf

[crf370137-bib-0051] FAO . (2023). Suite of food security indicators. FAOSTAT. https://www.fao.org/faostat/en/#data/FS

[crf370137-bib-0198] Franz, C. M. , Huch, M. , Mathara, J. M. , Abriouel, H. , Benomar, N. , Reid, G. , Galvez, A. & Holzapfel, W. H. (2014). African fermented foods and probiotics. International Journal of Food Microbiology, 190, 84–96. 10.1016/j.ijfoodmicro.2014.08.033 25203619

[crf370137-bib-0052] Farah, Z. , Mollet, M. , Younan, M. , & Dahir, R. (2007). Camel dairy in Somalia: Limiting factors and development potential. Livestock Science, 110(1‐2), 187–191. 10.1016/j.livsci.2006.12.010

[crf370137-bib-0053] Flibert, G. , Abel, T. , & Aly, S. (2016). African cassava traditional fermented food: The microorganisms' contribution to their nutritional and safety values—A review. International Journal of Current Microbiology and Applied Sciences, 5(10), 664–687.

[crf370137-bib-0054] Francis, J. M. , Grosskurth, H. , Kapiga, S. H. , Weiss, H. A. , Mwashiuya, J. , & Changalucha, J. (2017). Ethanol concentration of traditional alcoholic beverages in Northern Tanzania. Journal of Studies on Alcohol and Drugs, 78(3), 476–477. 10.15288/jsad.2017.78.476 28499118

[crf370137-bib-0056] Gebre, G. G. (2021). Prevalence of household food insecurity in East Africa: Linking food access with climate vulnerability. Climate Risk Management, 33, 100333. 10.1016/j.crm.2021.100333 PMC8390380 34476175

[crf370137-bib-0057] Gebre, T. S. , Emire, S. A. , Aloo, S. O. , Chelliah, R. , Vijayalakshmi, S. , & Oh, D. H. (2024). Unveiling the potential of African fermented cereal‐based beverages: Probiotics, functional drinks, health benefits, and bioactive components. Food Research International, 164, 114656. 10.1016/j.foodres.2024.114656 39059934

[crf370137-bib-0058] Getnet, B. , & Berhanu, A. (2016). Microbial dynamics, roles, and physicochemical properties of ‘Korefe’, a traditional fermented Ethiopian beverage. Biotechnology International, 9, 56–175.

[crf370137-bib-0059] Ghosh, S. , Bornman, C. , Meskini, M. , & Joghataei, M. (2024). Microbial diversity in African foods and beverages: A systematic assessment. Current Microbiology, 81(1), 19. 10.1007/s00284-024-02952-7 PMC10678836 38008849

[crf370137-bib-0060] Giraffa, G. , Chanishvili, N. , & Widyastuti, Y. (2010). Importance of lactobacilli in food and feed biotechnology. Research in Microbiology, 161(6), 480–487. 10.1016/j.resmic.2010.03.001 20302928

[crf370137-bib-0061] Girma, A. , & Aemiro, A. (2021). Antibacterial activity of lactic acid bacteria isolated from fermented Ethiopian traditional dairy products against food spoilage and pathogenic bacterial strains. Journal of Food Quality, 2021, 9978561. 10.1155/2021/9978561

[crf370137-bib-0062] Girma, T. , Bultosa, G. , & Bussa, N. (2013). Effect of grain tef [*Eragrostis tef* (Zucc.) Trott] flour substitution with flaxseed on quality and functionality of injera. International Journal of Food Science & Technology, 48(2), 350–356. 10.1111/j.1365-2621.2012.03194.x

[crf370137-bib-0063] Gonfa, A. , Foster, H. A. , & Holzapfel, W. H. (2001). Field survey and literature review on traditional fermented milk products of Ethiopia. International Journal of Food Microbiology, 68(3), 173–186. 10.1016/S0168-1605(01)00492-5 11529440

[crf370137-bib-0064] Greiner, C. , & Sakdapolrak, P. (2013). Rural–urban migration, agrarian change, and the environment in Kenya: A critical review of the literature. Population and Environment, 34(4), 524–553. 10.1007/s11111-013-0204-1

[crf370137-bib-0065] Guan, N. , & Liu, L. (2020). Microbial response to acid stress: Mechanisms and applications. Applied Microbiology and Biotechnology, 104(1), 51–65. 31773206 10.1007/s00253-019-10226-1 PMC6942593

[crf370137-bib-0066] Hailu, Y. , Hansen, E. B. , Seifu, E. , Eshetu, M. , Ipsen, R. , & Kappeler, S. (2016). Functional and technological properties of camel milk proteins: A review. Journal of Dairy Research, 83(4), 422–429. 27845026 10.1017/S0022029916000686

[crf370137-bib-0067] Hasan, M. N. , Sultan, M. Z. , & Mar‐E‐Um, M. (2014). Significance of fermented food in nutrition and food science. Journal of Scientific Research, 6(2), 373–386.

[crf370137-bib-0068] Hazra, T. , & Lodh, J. (2023). Ghee: Chemistry, technology, and health aspects. CRC Press.

[crf370137-bib-0069] Holzapfel, W. H. (2002). Appropriate starter culture technologies for small‐scale fermentation in developing countries. International Journal of Food Microbiology, 75(3), 197–212. 10.1016/S0168-1605(01)00707-3 12036143

[crf370137-bib-0070] Hotessa, N. , & Robe, J. (2020). Ethiopian indigenous traditional fermented beverage: The role of microorganisms toward nutritional and safety value of fermented beverage. International Journal of Microbiology, 2020(1), 8891259. 10.1155/2020/8891259 33488731 PMC7803167

[crf370137-bib-0071] Hunduma, T. , & Ashenafi, M. (2011). Effect of altitude on microbial succession during traditional enset (*Ensete ventricosum*) fermentation. International Journal of Food, Nutrition and Public Health, 4(1), 39–51.

[crf370137-bib-0072] Hussien, B. , Hailu, Y. , & Eshetu, M. (2021). Physicochemical properties and microbiological quality of *ititu* (traditionally fermented cow milk) in selected districts of Borena Zone, Oromia Regional State, Ethiopia. Open Journal of Animal Sciences, 11(2), 125–138.

[crf370137-bib-0073] Irakoze, M. L. , Wafula, E. N. , & Owaga, E. (2021). Potential role of African fermented indigenous vegetables in maternal and child nutrition in sub‐Saharan Africa. International Journal of Food Science, 2021(1), 3400329. 10.1155/2021/3400329 34957295 PMC8695012

[crf370137-bib-0075] Isono, Y. , Shingu, I. , & Shimizu, S. (1994). Identification and characteristics of lactic acid bacteria isolated from Masai fermented milk in Northern Tanzania. Bioscience, Biotechnology, and Biochemistry, 58(4), 660–664. 10.1271/bbb.58.660

[crf370137-bib-0076] Jans, C. , Meile, L. , Kaindi, D. W. M. , Kogi‐Makau, W. , Lamuka, P. , Renault, P. , Kreikemeyer, B. , Lacroix, C. , Hattendorf, J. , Zinsstag, J. , Schelling, E. , Fokou, G. , & Bonfoh, B. (2017). African fermented dairy products—Overview of predominant technologically important microorganisms focusing on African *Streptococcus infantarius* variants and potential future applications for enhanced food safety and security. International Journal of Food Microbiology, 250, 27–36. 10.1016/j.ijfoodmicro.2017.03.012 28364623

[crf370137-bib-0077] Jespersen, L. (2003). Occurrence and taxonomic characteristics of strains of *Saccharomyces cerevisiae* predominant in African indigenous fermented foods and beverages. FEMS Yeast Research, 3(2), 191–200. 10.1016/S1567-1356(02)00185-X 12702452

[crf370137-bib-0078] Johansen, P. G. , Owusu‐Kwarteng, J. , Parkouda, C. , Padonou, S. W. , & Jespersen, L. (2019). Occurrence and importance of yeasts in indigenous fermented food and beverages produced in sub‐Saharan Africa. Frontiers in Microbiology, 10, 1789. 31447811 10.3389/fmicb.2019.01789 PMC6691171

[crf370137-bib-0079] Joye, I. (2019). Protein digestibility of cereal products. Foods, 8(6), 199. 10.3390/foods8060199 31181787 PMC6617089

[crf370137-bib-0080] Karssa, T. H. , Kussaga, J. B. , Semedo‐Lemsaddek, T. , & Mugula, J. K. (2024). Insights on the microbiology of Ethiopian fermented milk products: A review. Food Science & Nutrition, 12(10), 6990–7003. 39479617 10.1002/fsn3.4372 PMC11521749

[crf370137-bib-0081] Kassa, B. , & Seifu, E. (2012). Physicochemical properties and microbiological quality of Dhanaan: Traditional fermented camel milk produced in eastern Ethiopia. [MSc thesis, School of Animal and Range Sciences, Haramaya University].

[crf370137-bib-0082] Katongole, J. N. (2008). *The microbial succession in indigenous fermented maize products* [Doctoral dissertation. University of the Free State]. http://hdl.handle.net/11660/1065

[crf370137-bib-0084] Kimaryo, V. M. , Massawe, G. A. , Olasupo, N. A. , & Holzapfel, W. H. (2000). The use of a starter culture in the fermentation of cassava for the production of “kivunde,” a traditional Tanzanian food product. International Journal of Food Microbiology, 56(2‐3), 179–190. 10.1016/S0168-1605(00)00159-8 10857544

[crf370137-bib-0085] Kitabatake, N. , Gimbi, D. M. , & Oi, Y. (2003). Traditional non‐alcoholic beverage, Togwa, in East Africa, produced from maize flour and germinated finger millet. International Journal of Food Sciences and Nutrition, 54(6), 447–455. 10.1080/09637480120092053 14522690

[crf370137-bib-0086] Kitessa, D. A. , Bacha, K. , Tola, Y. B. , & Murimi, M. (2022). Preparation and consumption of Shameta: An indigenous cereal‐based fermented porridge in Western Ethiopia. East African Journal of Sciences, 17(1), 55–70. 10.20372/eajs.v17i1.1962

[crf370137-bib-0087] Kohajdová, Z. (2017). Fermented cereal products. In A. Pandey , M. Á. Sanromán , G. Du , C. R. Soccol , C.‐D. Dussap (Eds.), Current developments in biotechnology and bioengineering (pp. 91–117). Elsevier. 10.1016/B978-0-444-63666-9.00004-2

[crf370137-bib-0088] Korir, S. C. , Imungi, J. K. , & Muroki, N. M. (1998). Proximate chemical composition of street foods and their energy and protein contribution to the nutrition of manual workers in Nairobi. Ecology of Food and Nutrition, 37(2), 123–133. 10.1080/03670244.1998.9991541

[crf370137-bib-0089] Kotala, J. , & Onyango, E. (2015). *Lactobacillus rhamnosus* cultured milk isolate may reduce adipogenesis. FASEB Journal, 29, 1010–1013. 10.1096/fasebj.29.1_supplement.1010.3

[crf370137-bib-0090] Kris‐Etherton, P. , Fleming, J. , & Harris, W. S. (2010). The debate about n‐6 polyunsaturated fatty acid recommendations for cardiovascular health. Journal of the American Dietetic Association, 110(2), 201–204. 10.1016/j.jada.2009.12.006 20102846

[crf370137-bib-0091] Kubo, R. (2014). Production of indigenous alcoholic beverages in a rural village of Tanzania. Journal of the Institute of Brewing, 120(2), 142–148. 10.1002/jib.127

[crf370137-bib-0092] Kubo, R. , & Kilasara, M. (2016). Brewing technique of *Mbege*, a banana beer produced in Northeastern Tanzania. Beverages, 2(3), 21. 10.3390/beverages2030021

[crf370137-bib-0093] Kumitch, H. M. (2019). *The effect of solid‐state fermentation on air‐classified pea protein‐enriched flour to improve the digestibility and functional properties* [Doctoral dissertation, University of Saskatchewan].

[crf370137-bib-0094] Kunyanga, C. N. , Mbugua, S. K. , Kangethe, E. K. , & Imungi, J. K. (2009). Microbiological and acidity changes during the traditional production of kirario: An indigenous Kenyan fermented porridge produced from green maize and millet. African Journal of Food, Agriculture, Nutrition and Development, 9(6). 10.4314/ajfand.v9i6.46261

[crf370137-bib-0095] Kyawt, Y. Y. , Imai, Y. , Yara, T. , & Kawamoto, Y. (2014). Effect of ensiling process and additive effects of fermented juice of epiphytic lactic acid bacteria on the cyanide content of two varieties of cassava. Animal Nutrition and Feed Technology, 14(3), 447–460. 10.5958/0974-181X.2014.01347.X

[crf370137-bib-0097] Lorri, W. , & Svanberg, U. (1995). An overview of the use of fermented foods for child feeding in Tanzania. Ecology of Food and Nutrition, 34(1), 65–81. 10.1080/03670244.1995.9991448

[crf370137-bib-0099] Lyumugabe, F. , Gros, J. , Nzungize, J. , Bajyana, E. , & Thonart, P. (2012). Characteristics of African traditional beers brewed with sorghum malt: A review. Biotechnologie, Agronomie, Société Et Environnement, 16(4). https://hdl.handle.net/2268/142889

[crf370137-bib-0100] Makokha, A. O. , Oniang'o, R. K. , Njoroge, S. M. , & Kamar, O. K. (2002). Effect of traditional fermentation and malting on phytic acid and mineral availability from sorghum (*Sorghum bicolor*) and finger millet (*Eleusine coracana*) grain varieties grown in Kenya. Food and Nutrition Bulletin, 23(3), 241–245. 10.1177/15648265020233S147 12362804

[crf370137-bib-0101] Mamo, J. , Kumera, B. , & Asmamaw, M. (2016). Evaluation of microbiological quality of raw milk, homemade Ergo and homemade Ayib in North Shoa District, Amhara, Ethiopia. Pakistan Journal of Food Science, 26(2), 83–91.

[crf370137-bib-0102] Marco, M. L. , Heeney, D. , Binda, S. , Cifelli, C. J. , Cotter, P. D. , Foligné, B. , & O'Sullivan, O. (2017). Health benefits of fermented foods: Microbiota and beyond. Current Opinion in Biotechnology, 44, 94–102. 10.1016/j.copbio.2017.02.003 27998788

[crf370137-bib-0103] Marcus, H. G. , & Low, D. A. (2023). *Eastern Africa*. Encyclopedia Britannica. https://www.britannica.com/place/eastern‐Africa

[crf370137-bib-0104] Marshall, E. , & Mejia, D. (2011). Traditional fermented food and beverages for improved livelihoods. FAO Diversification Booklet. Food and Agriculture Organization of the United Nations (FAO).

[crf370137-bib-0105] Masha, G. G. K. , Ipsen, R. , Petersen, M. A. , & Jakobsen, M. (1998). Microbiological, rheological and aromatic characteristics of fermented Uji (an East African sour porridge). World Journal of Microbiology and Biotechnology, 14, 451–456. 10.1023/A:1008889900944

[crf370137-bib-0106] Materia, V. C. , Linnemann, A. R. , Smid, E. J. , & Schoustra, S. E. (2021). Upscaling of traditional fermented foods to build value chains and to promote women entrepreneurship . International Fund for Agricultural Development (IFAD). https://www.ifad.org/en/rural‐development‐report/#group‐section‐

[crf370137-bib-0107] Mathara, J. M. (1999). Studies on lactic acid producing microflora in mursik and kule naoto, traditional fermented milks from Nandi and Masai communities in Kenya. [Master's thesis, University of Nairobi].

[crf370137-bib-0108] Mathara, J. M. , Schillinger, U. , Kutima, P. M. , Mbugua, S. K. , & Holzapfel, W. H. (2004). Isolation, identification and characterization of the dominant microorganisms of *kule naoto*: The Maasai traditional fermented milk in Kenya. International Journal of Food Microbiology, 94(3), 269–278. 10.1016/j.ijfoodmicro.2004.01.008 15246238

[crf370137-bib-0109] Mattiello, S. , Caroprese, M. , Matteo, C. G. , Fortina, R. , Martini, A. , Martini, M. , Parisi, G. , Russo, C. , Zecchini, M. , & ASPA Commission “Animal Productions in Development Cooperation Projects” . (2018). Typical dairy products in Africa from local animal resources. Italian Journal of Animal Science, 17(3), 740–754. 10.1080/1828051X.2017.1401910

[crf370137-bib-0110] Misci, C. , Taskin, E. , Dall'Asta, M. , Fontanella, M. C. , Bandini, F. , Imathiu, S. , Parisi, G. , Russo, C. , & Zecchini, M. (2021). Fermentation as a tool for increasing food security and nutritional quality of indigenous African leafy vegetables: The case of *Cucurbita* sp. Food Microbiology, 99, 103820. 10.1016/j.fm.2021.103820 34119105

[crf370137-bib-0111] Misihairabgwi, J. M. , Ishola, A. , Quaye, I. , Sulyok, M. , & Krska, R. (2018). Diversity and fate of fungal metabolites during the preparation of *oshikundu*, a Namibian traditional fermented beverage. World Mycotoxin Journal, 11(3), 471–481. 10.3920/WMJ2018.2352

[crf370137-bib-0112] Miyamoto, M. , Nakajima, H. , Muigai, C. W. , Kiiyukia, C. , & Miyamoto, T. (2005). Identification of lactic acid bacteria isolated from Kenyan traditional fermented milk, Maziwa lala, and their symbiotic relationship. Milchwissenschaft, 60(4), 416–418. https://www.cabidigitallibrary.org/doi/full/10.5555/20053169869

[crf370137-bib-0113] Mohammed, N. A. , Ahmed, I. A. M. , & Babiker, E. E. (2011). Nutritional evaluation of sorghum flour (*Sorghum bicolor* L. Moench) during processing of injera. World Academy of Science, Engineering and Technology, 51, 72–76. 10.5281/zenodo.1083235

[crf370137-bib-0114] Mokua, R. A. (2004). *Effect of Kenyan fermented milk on Escherichia coli* [Master's thesis, University of Wisconsin‐Stout].

[crf370137-bib-0115] Montagnac, J. A. , Davis, C. R. , & Tanumihardjo, S. A. (2009). Nutritional value of cassava for use as a staple food and recent advances for improvement. Comprehensive Reviews in Food Science and Food Safety, 8(3), 181–194. 10.1111/j.1541-4337.2009.00077.x 33467798

[crf370137-bib-0116] Moonga, H. B. , Schoustra, S. E. , Linnemann, A. R. , Shindano, J. , & Smid, E. J. (2022). Towards valorisation of indigenous traditional fermented milk: Mabisi as a model. Current Opinion in Food Science, 46, 100835.

[crf370137-bib-0117] Moonga, H. B. , Schoustra, S. E. , van den Heuvel, J. , Linnemann, A. R. , Samad, M. S. , Shindano, J. , & Smid, E. J. (2020b). Composition and diversity of natural bacterial communities in mabisi, a traditionally fermented milk. Frontiers in Microbiology, 11, 1816. 10.3389/fmicb.2020.01816 32849423 PMC7406715

[crf370137-bib-0118] Motarjemi, Y. (2002). Impact of small scale fermentation technology on food safety in developing countries. International Journal of Food Microbiology, 75(3), 213–229. 10.1016/S0168-1605(01)00709-7 12036144

[crf370137-bib-0119] Mugula, J. K. , Nnko, S. A. M. , Narvhus, J. A. , & Sørhaug, T. (2003). Microbiological and fermentation characteristics of *togwa*, a Tanzanian fermented food. International Journal of Food Microbiology, 80(3), 187–199. 10.1016/S0168-1605(02)00141-1 12423921

[crf370137-bib-0120] Mugula, J. K. , Nnko, S. A. M. , & Sørhaug, T. (2001). Changes in quality attributes during storage of *togwa*, a lactic acid fermented gruel. Journal of Food Safety, 21(3), 181–194. 10.1111/j.1745-4565.2001.tb00317.x

[crf370137-bib-0121] Muigei, S. C. , Shitandi, A. , Muliro, P. , & Bitonga, O. R. (2013). Production of exopolysaccharides in the Kenyan fermented milk, Mursik. International Journal of Scientific Research, 2(12), 79–89. http://41.89.96.81:8080/xmlui/handle/123456789/2712

[crf370137-bib-0122] Mukisa, I. M. , Nsiimire, D. G. , Byaruhanga, Y. B. , Muyanja, C. M. B. K. , Langsrud, T. , & Narvhus, J. A. (2010). Obushera: Descriptive sensory profiling and consumer acceptability. Journal of Sensory Studies, 25, 190–214. 10.1111/j.1745-459X.2009.00272.x

[crf370137-bib-0123] Mukisa, I. M. , Porcellato, D. , Byaruhanga, Y. B. , Muyanja, C. M. , Rudi, K. , Langsrud, T. , & Narvhus, J. A. (2012). The dominant microbial community associated with fermentation of *Obushera* (sorghum and millet beverages) determined by culture‐dependent and culture‐independent methods. International Journal of Food Microbiology, 160(1), 1–10. 10.1016/j.ijfoodmicro.2012.09.023 23141639

[crf370137-bib-0124] Mukisa, I. M. , Ssendagala, G. W. , & Byakika, S. (2020). Microbiological safety and physicochemical composition of *Bongo*, a traditional fermented milk product from Lyantonde district, Uganda. Scientific African, 10, e00583. 10.1016/j.sciaf.2020.e00583

[crf370137-bib-0125] Mulaw, G. , Muleta, D. , Tesfaye, A. , & Sisay, T. (2020). Protective effect of potential probiotic strains from fermented Ethiopian food against *Salmonella Typhimurium* DT104 in mice. International Journal of Microbiology, 2020, 7523629. 10.1155/2020/7523629 32351574 PMC7178517

[crf370137-bib-0126] Mulaw, G. , Sisay Tessema, T. , Muleta, D. , & Tesfaye, A. (2019). In vitro evaluation of probiotic properties of lactic acid bacteria isolated from some traditionally fermented Ethiopian food products. International Journal of Microbiology, 2019, 7179514. 10.1155/2019/7179514 31534458 PMC6732631

[crf370137-bib-0127] Mulaw, G. , & Tesfaye, A. (2017). Technology and microbiology of traditionally fermented food and beverage products of Ethiopia: A review. African Journal of Microbiology Research, 11(21), 825–844.

[crf370137-bib-0128] Muyanja, C. M. B. K. , Birungi, S. , Ahimbisibwe, M. , Semanda, J. , & Namugumya, B. S. (2010). Traditional processing, microbial and physicochemical changes during fermentation of *malwa* . African Journal of Food, Agriculture, Nutrition and Development, 10(10). 10.4314/ajfand.v10i10.62891

[crf370137-bib-0129] Muyanja, C. M. B. K. , Kikafunda, J. K. , Narvhus, J. A. , Helgetun, K. , & Langsrud, T. (2003). Production methods and composition of Bushera: A Ugandan traditional fermented cereal beverage. African Journal of Food, Agriculture, Nutrition and Development, 3(1), 10–19. 10.4314/ajfand.v3i1.19108

[crf370137-bib-0130] Muyanja, C. M. B. K. , & Namugumya, B. S. (2009). Traditional processing, microbiological, physicochemical and sensory characteristics of kwete, a Ugandan fermented maize‐based beverage. African Journal of Food, Agriculture, Nutrition and Development, 9(4), 1046‐1049. 10.4314/ajfand.v9i4.43876

[crf370137-bib-0131] Muyanja, C. M. B. K. , Narvhus, J. A. , Treimo, J. , & Langsrud, T. (2003). Isolation, characterization and identification of lactic acid bacteria from bushera: A Ugandan traditional fermented beverage. International Journal of Food Microbiology, 80(3), 201–210. 10.1016/S0168-1605(02)00148-4 12494920

[crf370137-bib-0132] Muyonga, J. H. , Aworh, O. C. , Kinyuru, J. , Manley, M. , Nansereko, S. , & Nyangena, D. N. (2018). Nutritional and nutraceutical properties of traditional African foods. In Public health, disease and development in Africa (pp. 229–244). Routledge.

[crf370137-bib-0133] Mwizerwa, H. , Abong, G. O. , Mbugua, S. K. , Okoth, M. W. , Gacheru, P. , & Muiru, M. (2018). Profiling of microbial content and growth in fermented maize‐based products from Western Kenya. Current Research in Nutrition and Food Science Journal, 6(2), 509–519. 10.12944/CRNFSJ.6.2.25

[crf370137-bib-0134] Nakamura, T. , Sugai, M. , Nakamura, A. , Ariga, H. , Koaze, H. , Kiiyukia, C. , & Urashima, T. (1999). Microbiological properties of Maziwa lala, a Kenyan traditional fermented milk of the Masai community in Kenya. Milk Science, 48(1), 9–14.

[crf370137-bib-0135] Nakavuma, J. L. , Møller, P. L. , Jakobsen, M. , Salimo, P. , & Nasinyama, G. W. (2012). Processing steps and lactic acid bacteria involved in traditional cultured milk (Kwerionik) production in Uganda. Africa Journal of Animal and Biomedica Sciences, 7(2), 82–94.

[crf370137-bib-0136] Nakuwa, M. S. , Mongi, R. , & Ngoma, S. (2023). Food handling practices, the prevalence of aflatoxin dietary exposure, and its associated factors among children aged 6–23 months in Bukombe District, Tanzania. NFS Journal, 31, 162–170. 10.1016/j.nfs.2022.12.007

[crf370137-bib-0137] Nduko, J. M. , Matofari, J. W. , Nandi, Z. O. , & Sichangi, M. B. (2016). Spontaneously fermented Kenyan milk products: A review of the current state and future perspectives. African Journal of Food Science, 11(1), 1–11. http://41.89.96.81:8080/xmlui/handle/123456789/2713

[crf370137-bib-0138] Neela, S. , & Fanta, S. W. (2020). Injera (An ethnic, traditional staple food of Ethiopia): A review on traditional practice to scientific developments. Journal of Ethnic Foods, 7(1), 32. 10.1186/s42779-020-00069-x

[crf370137-bib-0139] Nemo, R. , & Bacha, K. (2021). Natural preservative‐based shelf‐life enhancement of borde: A traditional Ethiopian low alcoholic beverage. Journal of Food Processing and Preservation, 45(11), e15968. 10.1111/jfpp.15968

[crf370137-bib-0140] Nielsen, D. S. , Cho, G. S. , Hanak, A. , Huch, M. , Franz, C. M. , & Arneborg, N. (2010). The effect of bacteriocin‐producing *Lactobacillus plantarum* strains on the intracellular pH of sessile and planktonic *Listeria monocytogenes* single cells. International Journal of Food Microbiology, 141, S53–S59. 10.1016/j.ijfoodmicro.2010.03.040 20447709

[crf370137-bib-0141] Nieminen, M. T. , Novak‐Frazer, L. , Collins, R. , Dawsey, S. P. , Dawsey, S. M. , Abnet, C. C. , White, R. E. , Freedman, N. D. , Mwachiro, M. , Bowyer, P. , Salaspuro, M. , & Rautemaa, R. (2013). Alcohol and acetaldehyde in African fermented milk mursik—A possible etiologic factor for high incidence of esophageal cancer in western Kenya. Cancer Epidemiology, Biomarkers & Prevention, 22(1), 69–75. 10.1158/1055-9965.EPI-12-0908 PMC3538938 23155139

[crf370137-bib-0142] Nigatu, A. , & Gashe, B. A. (1994). Survival and growth of selected pathogens in fermented kocho (*Ensete ventricosum*). East African Medical Journal, 71(8), 514–518. 7867544

[crf370137-bib-0143] Njage, K. M. P. , & Wangoh, J. (2008). Impact of the lactoperoxidase system on activity of selected lactic starter cultures in camel milk. Food, 14, 70–74. http://erepository.uonbi.ac.ke:8080/xmlui/handle/123456789/36146

[crf370137-bib-0145] Nyambane, B. , Thari, W. M. , Wangoh, J. , & Njage, P. M. (2014). Lactic acid bacteria and yeasts involved in the fermentation of *amabere amaruranu*, a Kenyan fermented milk. Food Science & Nutrition, 2(6), 692–699. 10.1002/fsn3.162 25493187 PMC4256574

[crf370137-bib-0146] Nyanzi, R. , & Jooste, P. J. (2012). Cereal‐based functional foods. In E. C. Rigobelo (Eds.), Probiotics (pp. 161–197). IntechOpen.

[crf370137-bib-0147] Obafemi, Y. D. , Oranusi, S. U. , Ajanaku, K. O. , Akinduti, P. A. , Leech, J. , & Cotter, P. D. (2022). African fermented foods: Overview, emerging benefits, and novel approaches to microbiome profiling. Npj Science of Food, 6(1), 15. 10.1038/s41538-022-00090-2 35181677 PMC8857253

[crf370137-bib-0148] Oi, Y. , & Kitabatake, N. (2003). Chemical composition of an East African traditional beverage, togwa. Journal of Agricultural and Food Chemistry, 51(24), 7024–7028. 14611165 10.1021/jf0203343

[crf370137-bib-0149] Okoit, S. (2022). Investigation of aflatoxins in a traditional brew of malwa and obushera in Uganda and development of an identification kit for these aflatoxins in the above foodstuffs [Doctoral dissertation, Busitema University]. http://hdl.handle.net/20.500.12283/1088

[crf370137-bib-0150] Omay, P. O. , Muthama, N. J. , Oludhe, C. , Kinama, J. M. , Artan, G. , & Atheru, Z. (2024). Trends of hunger and food insecurity over IGAD region of Eastern Africa. International Journal of Food, Nutrition, and Safety, 15(1), 1–21.

[crf370137-bib-0151] Onyango, C. (2014). Physical properties of dry‐milled maize meals and their relationship with the texture of stiff and thin porridge. African Journal of Food Science, 8(8), 435–443.

[crf370137-bib-0152] Owaga, E. , Muga, R. , Mumbo, H. , & Aila, F. (2011). Chronic dietary aflatoxins exposure in Kenya and emerging public health concerns of impaired growth and immune suppression in children. International Journal of Biological and Chemical Sciences, 5(3). 10.4314/ijbcs.v5i3.72287

[crf370137-bib-0153] Oyedeji, A. B. , Green, E. , Jeff‐Agboola, Y. A. , Olanbiwoninu, A. A. , Areo, E. , Martins, I. E. , El‐Imam, A. M. A., & Adebo, O. A. (2023). Presence of pathogenic microorganisms in fermented foods. In O. A. Adebo, A. A. Obadina, & S. K. Panda (Eds.), Indigenous fermented foods for the tropics (pp. 519–537). Academic Press.

[crf370137-bib-0154] Oyewole, M. F. (2016). *Effects of development partnership in higher education project on welfare status of rural women processors in Oyo and Osun states, Nigeria* [Doctoral dissertation, University of Ibadan].

[crf370137-bib-0155] Patel, K. , Guenther, D. , Wiebe, K. , & Seburn, R.‐A. (2014). Promoting food security and livelihoods for urban poor through the informal sector: A case study of street food vendors in Madurai, Tamil Nadu, India. Food Security, 6, 861–878. 10.1007/s12571-014-0391-1

[crf370137-bib-0156] Patel, K. , Wakhisi, J. , Mining, S. , Mwangi, A. , & Patel, R. (2013). Esophageal cancer, the topmost cancer at MTRH in the Rift Valley, Kenya, and its potential risk factors. ISRN Oncol, 2013(9), 503249. 24490085 10.1155/2013/503249 PMC3893746

[crf370137-bib-0157] Penna, A. L. B. , Paula, A. T. , Casarotti, S. N. , Diamantino, V. , & Silva, L. (2015). Overview of the functional lactic acid bacteria in the fermented milk products. In V. R. Rai , J. A. Bai (Eds.), Beneficial microbes in fermented and functional foods (pp. 100–154). CRC Press.

[crf370137-bib-0158] Pinto, M. G. V. , Franz, C. M. , Schillinger, U. , & Holzapfel, W. H. (2006). *Lactobacillus* spp. with in vitro probiotic properties from human faeces and traditional fermented products. International Journal of Food Microbiology, 109(3), 205–214. 10.1016/j.ijfoodmicro.2006.01.029 16503361

[crf370137-bib-0159] Ponnampalam, E. N. , Priyashantha, H. , Vidanarachchi, J. K. , Kiani, A. , & Holman, B. W. (2024). Effects of nutritional factors on fat content, fatty acid composition, and sensorial properties of meat and milk from domesticated ruminants: An overview. Animals, 14(6), 840. 38539939 10.3390/ani14060840 PMC10967350

[crf370137-bib-0160] Pratiwi, C. , Rahayu, W. P. , Lioe, H. N. , Herawati, D. , Broto, W. , & Ambarwati, S. (2015). The effect of temperature and relative humidity on *Aspergillus flavus* BIO 2237 growth and aflatoxin production on soybeans. International Food Research Journal, 22(1), 82–87.

[crf370137-bib-0162] Robert, O. J. (2011). Genetic analysis of Striga hermonthica resistance in sorghum (Sorghum bicolor) genotypes in Eastern Uganda [Doctoral dissertation, University of KwaZulu‐Natal].

[crf370137-bib-0164] Samoei, E. J. (2015). The influence of Kass FM in the promotion of food security among the Tugen in Perkerra Sub‐location: A case study of Marigat Division of Baringo County (Doctoral dissertation, University of Nairobi).

[crf370137-bib-0165] Samtiya, M. , Aluko, R. E. , & Dhewa, T. (2020). Plant food anti‐nutritional factors and their reduction strategies: An overview. Food Production, Processing and Nutrition, 2, 1–14. 10.1186/s43014-020-0020-5

[crf370137-bib-0166] Seifu, E. (2013). Chemical composition and microbiological quality of Metata Ayib: A traditional Ethiopian fermented cottage cheese. International Food Research Journal, 20(1), 93.

[crf370137-bib-0167] Shayo, N. B. , Kamala, A. , Gidamis, A. B. , & Nnko, S. A. M. (2000). Aspects of manufacture, composition, and safety of *orubisi*: A traditional alcoholic beverage in the north‐western region of Tanzania. International Journal of Food Sciences and Nutrition, 51(5), 395–402. 10.1080/096374800426993 11103305

[crf370137-bib-0168] Shayo, N. B. , Nnko, S. A. M. , & Gidamis, A. B. (1998). Isolation and characterisation of yeasts and bacteria from *Mbege*—An opaque beer made from millet malt and banana juice. Tanzania Journal of Agricultural Sciences, 1(1), 57–63.

[crf370137-bib-0170] Srinivas, M. , O'Sullivan, O. , Cotter, P. D. , van Sinderen, D. , & Kenny, J. G. (2022). The application of metagenomics to study microbial communities and develop desirable traits in fermented foods. Foods, 11(20), 3297. 10.3390/foods11203297 37431045 PMC9601669

[crf370137-bib-0171] Swelum, A. A. , El‐Saadony, M. T. , Abdo, M. , Ombarak, R. A. , Hussein, E. O. , Suliman, G. , & Abd El‐Hack, M. E. (2021). Nutritional, antimicrobial and medicinal properties of camel's milk: A review. Saudi Journal of Biological Sciences, 28(5), 3126–3136. 10.1016/j.sjbs.2021.02.057 34025186 PMC8117040

[crf370137-bib-0172] Tadesse, N. S. , Beyene, G. F. , Hordofa, T. B. , & Hailu, A. A. (2020). Traditional foods and beverages in Eastern Tigray of Ethiopia. Journal of Ethnic Foods, 7(1), 16. 10.1186/s42779-020-00050-8

[crf370137-bib-0173] Tamang, J. P. , Cotter, P. D. , Endo, A. , Han, N. S. , Kort, R. , Liu, S. Q. , & Hutkins, R. (2020). Fermented foods in a global age: East meets West. Comprehensive Reviews in Food Science and Food Safety, 19(1), 184–217. 10.1111/1541-4337.12520 33319517

[crf370137-bib-0174] Tamene, A. , Baye, K. , Kariluoto, S. , Edelmann, M. , Bationo, F. , Leconte, N. , & Humblot, C. (2019). *Lactobacillus plantarum* P2R3FA isolated from traditional cereal‐based fermented food increases folate status in deficient rats. Nutrients, 11(11), 2819. 10.3390/nu11112819 31752138 PMC6893693

[crf370137-bib-0175] Tarimo, C. B. , & Kaale, L. D. (2023). Use of yeasts in traditional alcoholic beverages in Tanzania and potential opportunities. Journal of the American Society of Brewing Chemists, 81(1), 1–11.

[crf370137-bib-0176] Tesfamariam, Y. , & Hurlbert, M. (2017). Gendered adaptation of Eritrean dryland farmers. International Journal of Climate Change Strategies and Management, 9(2), 207–224.

[crf370137-bib-0177] Tiruha Habte Karssa, T. H. K. , Kebede Abegaz Ali, K. A. A. , & Edessa Negera Gobena, E. N. G. (2014). The microbiology of kocho: An Ethiopian traditionally fermented food from enset (Ensete ventricosum). International Journal of Life Sciences, 8(1), 7–13.

[crf370137-bib-0178] Tusekwa, T. C. E. , Mosha, H. S. , Laswai, E. E. , & Towo, A. B. (2000). Traditional alcoholic beverages of Tanzania: Production, quality and changes in quality attributes during storage. International Journal of Food Sciences and Nutrition, 51(2), 135–143. 10.1080/096374800100831 10953757

[crf370137-bib-0180] Urga, K. , Fite, A. , & Biratu, E. (1997). Natural fermentation of enset (*Ensete ventricosum*) for the production of kocho. Ethiopian Journal of Health Development, 11(1), 1–7.

[crf370137-bib-0181] Uusiku, N. P. , Oelofse, A. , Duodu, K. G. , Bester, M. J. , & Faber, M. (2010). Nutritional value of leafy vegetables of sub‐Saharan Africa and their potential contribution to human health: A review. Journal of Food Composition and Analysis, 23(6), 499–509. 10.1016/j.jfca.2010.05.002

[crf370137-bib-0182] Valentino, V. , Magliulo, R. , Farsi, D. , Cotter, P. D. , O'Sullivan, O. , Ercolini, D. , & De Filippis, F. (2024). Fermented foods, their microbiome and its potential in boosting human health. Microbial Biotechnology, 17(2), e14428. 10.1111/1751-7915.14428 38393607 PMC10886436

[crf370137-bib-0183] Van Tienen, A. , Hullegie, Y. M. , Hummelen, R. , Hemsworth, J. , Changalucha, J. , & Reid, G. (2011). Development of a locally sustainable functional food for people living with HIV in Sub‐Saharan Africa: Laboratory testing and sensory evaluation. Beneficial Microbes, 2(3), 193–198. 21986358 10.3920/BM2011.0024

[crf370137-bib-0184] Vinderola, G. , Cotter, P. D. , Freitas, M. , Gueimonde, M. , Holscher, H. D. , Ruas‐Madiedo, P. , & O'Sullivan, O. (2023). Fermented foods: A perspective on their role in delivering biotics. Frontiers in Microbiology, 14, 1196239. 10.3389/fmicb.2023.1196239 37250040 PMC10213265

[crf370137-bib-0185] Wafula, E. N. , Franz, C. M. A. P. , Rohn, S. , Huch, M. , Mathara, J. M. , & Trierweiler, B. (2016). Fermentation of African indigenous leafy vegetables to lower post‐harvest losses, maintain quality and increase product safety. African Journal of Horticultural Science, 9, 1–13.

[crf370137-bib-0187] Wani, A. D. , Prasad, W. , Khamrui, K. , & Jamb, S. (2022). A review on quality attributes and utilization of ghee residue, an under‐utilized dairy by‐product. Future Foods, 5, 100131.

[crf370137-bib-0188] Wanjala, W. G. , Onyango, A. , Makayoto, M. , & Onyango, C. (2016). Indigenous technical knowledge and formulations of thick (ugali) and thin (uji) porridges consumed in Kenya. African Journal of Food Science, 10(12), 385–396. 10.5897/AJFS2016.1521

[crf370137-bib-0189] Weldemichael, H. , Stoll, D. , Weinert, C. , Berhe, T. , Admassu, S. , Alemu, M. , & Huch, M. (2019). Characterization of the microbiota and volatile components of kocho, a traditional fermented food of Ethiopia. Heliyon, 5(6). 10.1016/j.heliyon.2019.e01842 PMC6558307 31206089

[crf370137-bib-0190] WHO Estimates of the Global Burden of Foodborne Diseases . (2025). *Foodborne disease estimates*. https://www.who.int/teams/nutrition‐and‐food‐safety/monitoring‐nutritional‐status‐and‐food‐safety‐and‐events/foodborne‐disease‐estimates

[crf370137-bib-0191] Willis, J. (2002). Potent brews: A social history of alcohol in East Africa 1850–1999 . British Institute in Eastern Africa.

[crf370137-bib-0192] Woldemikael, T. M. (2009). Pitfalls of nationalism in Eritrea. In D. O'Kane & T. R. Hepner (Eds.), Biopolitics, militarism and development: Eritrea in the twenty‐first century (pp. 1–16). Berghahn Books.

[crf370137-bib-0193] Wolgamuth, E. , Yusuf, S. , Hussein, A. , & Pasqualone, A. (2022). A survey of laxoox/canjeero, a traditional Somali flatbread: Production styles. Journal of Ethnic Foods, 9(1), 22. 10.1186/s42779-022-00138-3

[crf370137-bib-0194] Wuyts, S. , Van Beeck, W. , Allonsius, C. N. , van den Broek, M. F. , & Lebeer, S. (2020). Applications of plant‐based fermented foods and their microbes. Current Opinion in Biotechnology, 61, 45–52. 10.1016/j.copbio.2019.09.023 31733464

[crf370137-bib-0195] Xu, J. , Guo, L. , Zhao, N. , Meng, X. , Zhang, J. , Wang, T. , & Fan, M. (2023). Response mechanisms to acid stress of acid‐resistant bacteria and biotechnological applications in the food industry. Critical Reviews in Biotechnology, 43(2), 258–274. 35114869 10.1080/07388551.2021.2025335

[crf370137-bib-0196] Yan, X. , McClements, D. J. , Luo, S. , Ye, J. , & Liu, C. (2024). A review of the effects of fermentation on the structure, properties, and application of cereal starch in foods. Critical Reviews in Food Science and Nutrition, Advance online publication. 10.1080/10408398.2024.2334269 38532611

[crf370137-bib-0197] Yegrem, L. , Abera, S. , & Temesgen, M. (2021). Nutritional composition and sensory quality of injera prepared from tef (*Eragrostis tef* (Zucc.) Trotter) complemented with lupine (*Lupinus* spp.). Cogent Food & Agriculture, 7(1), 1862469.

