# Supplementary figures and images for: Eastern African traditional fermented foods and beverages: Advancements, challenges, and perspectives on food technology, nutrition, and safety
Source: Compr Rev Food Sci Food Saf. 2025 Mar 4;24(2):e70137. doi: 10.1111/1541-4337.70137 (PMC11877266; doi:10.1111/1541-4337.70137)

## Slide 1
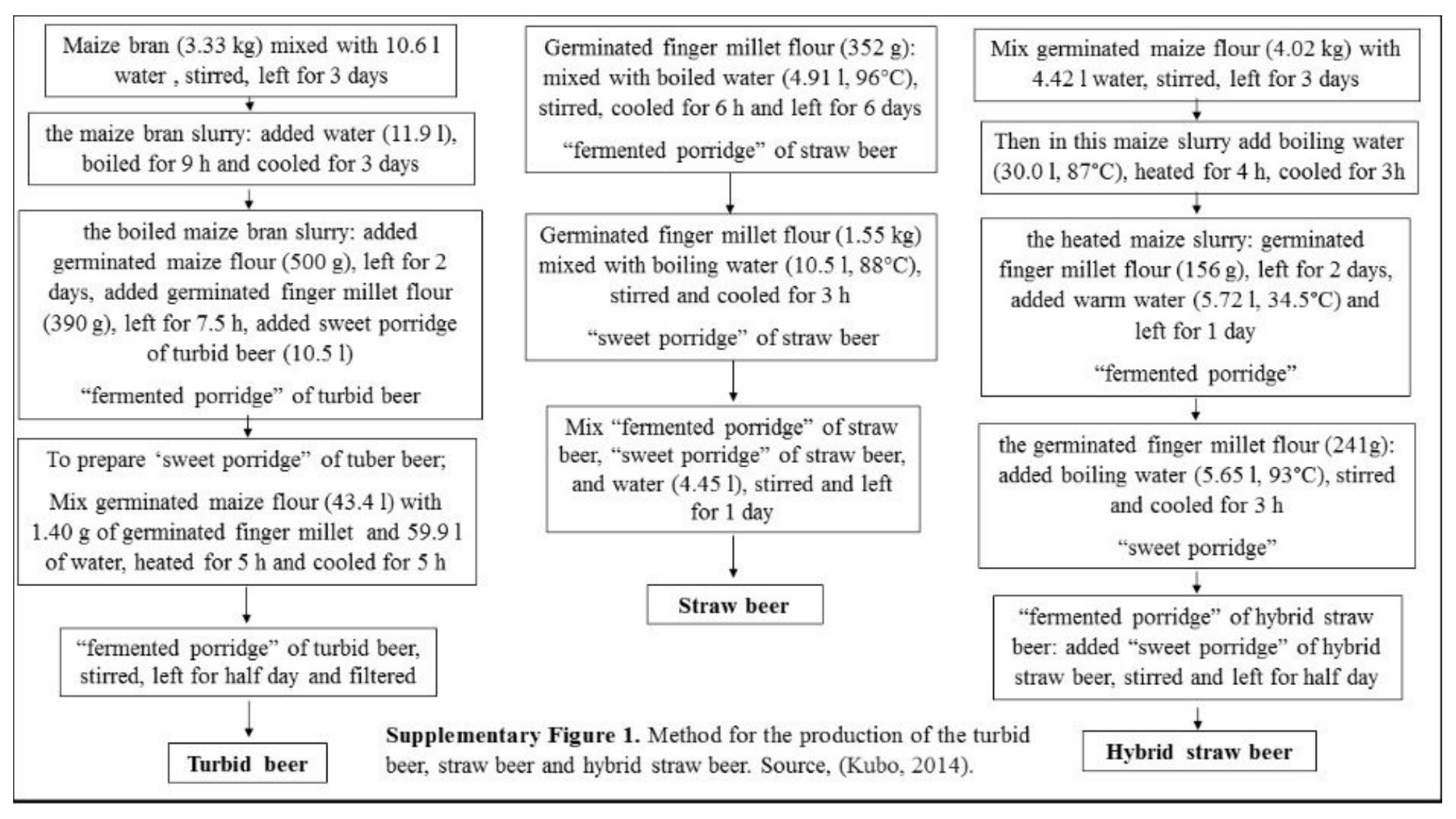

Supplement: Supplementary file 1 — Method for the production of the turbid beer, straw beer and hybrid straw beer. Source, (Kubo, 2014). [file CRF3-24-e70137-s002.pptx]

## Slide 1
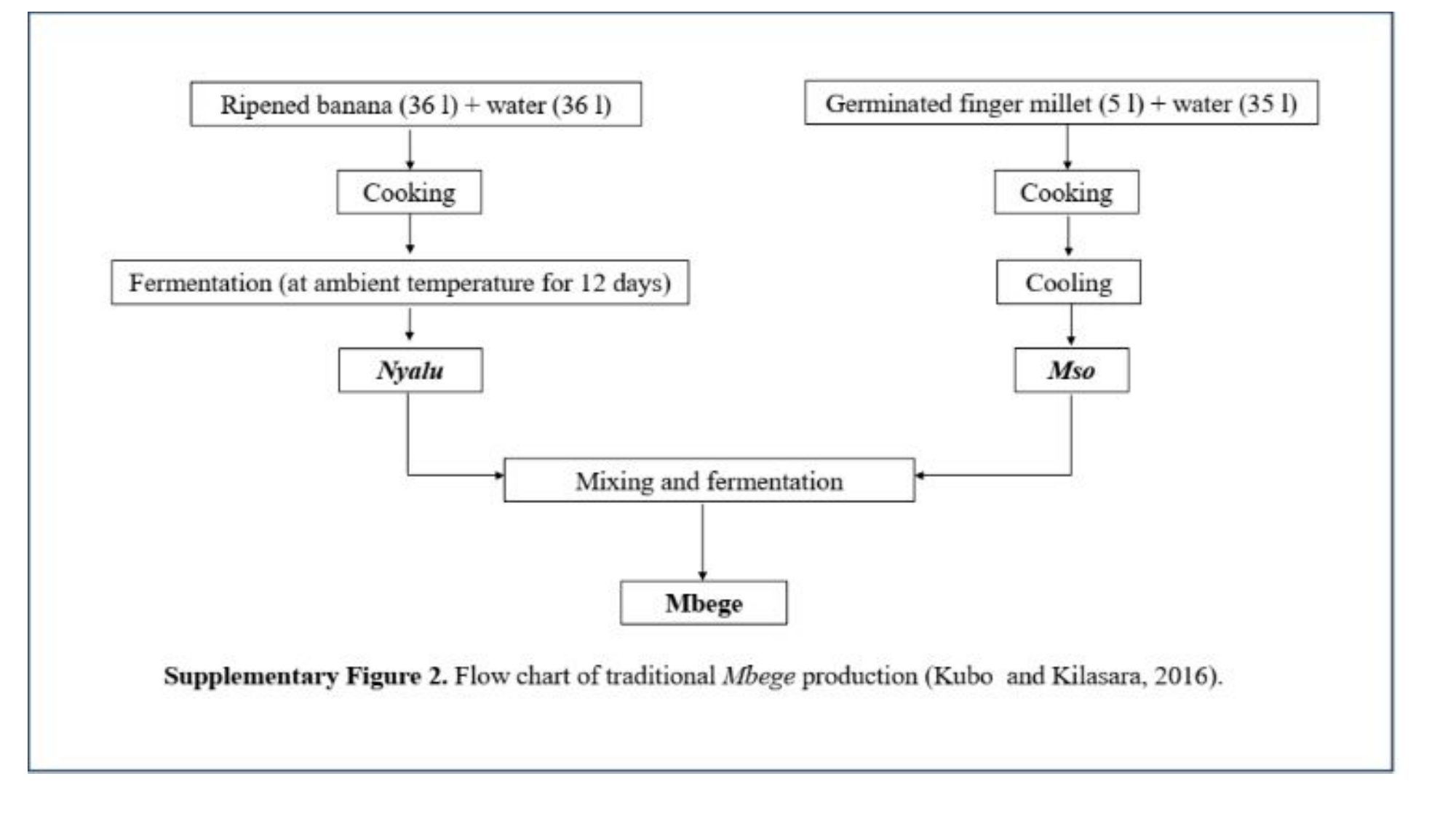

Supplement: Supplementary file 2 — Flow chart of traditional Mbege production Kubo and Kilasara 2016). [file CRF3-24-e70137-s001.pptx]

## Slide 1
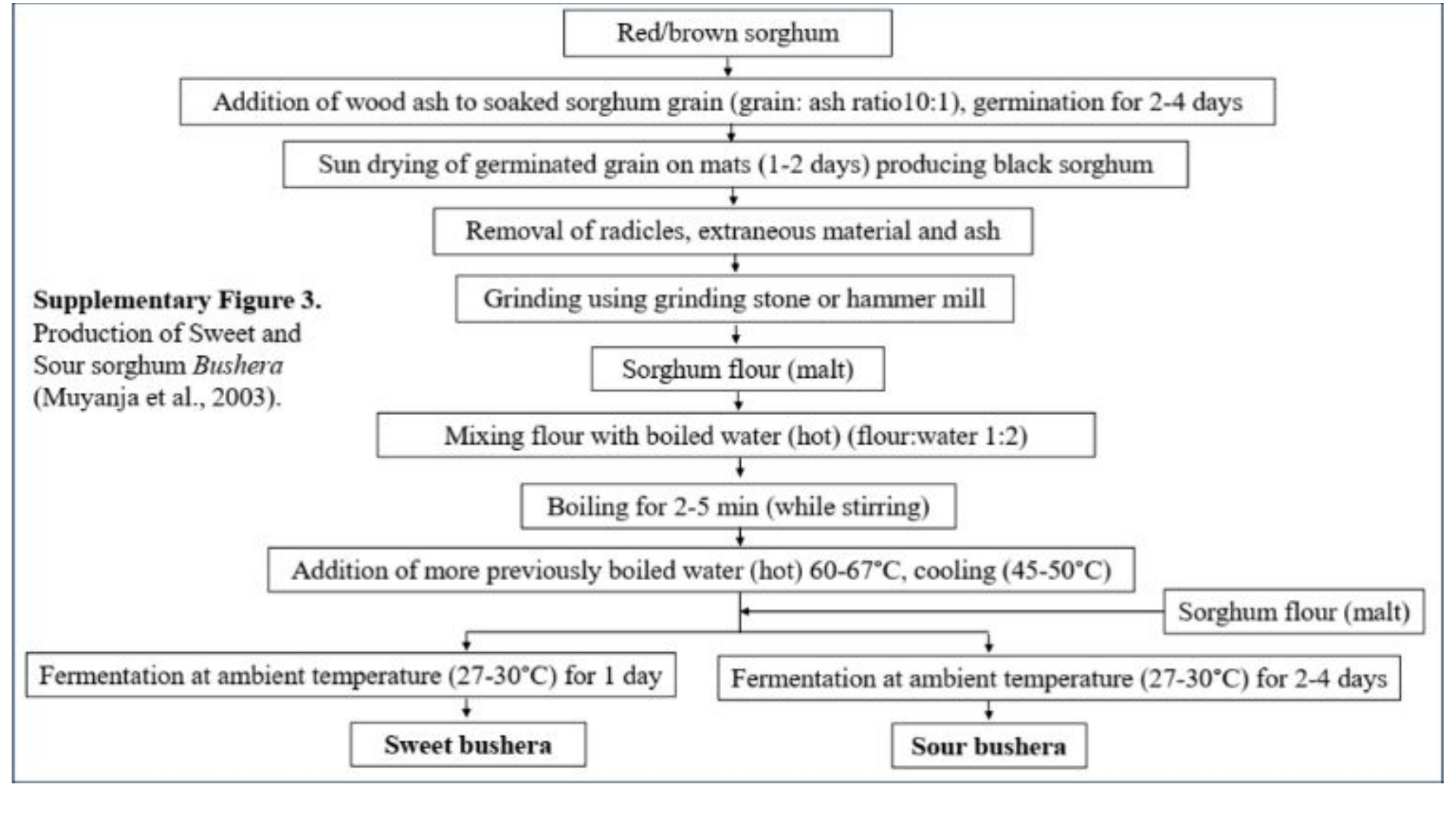

Supplement: Supplementary file 3 — Production of Sweet and Sour sorghum Bushera (Muyanja et aL., 2003). [file CRF3-24-e70137-s004.pptx]

## Slide 1
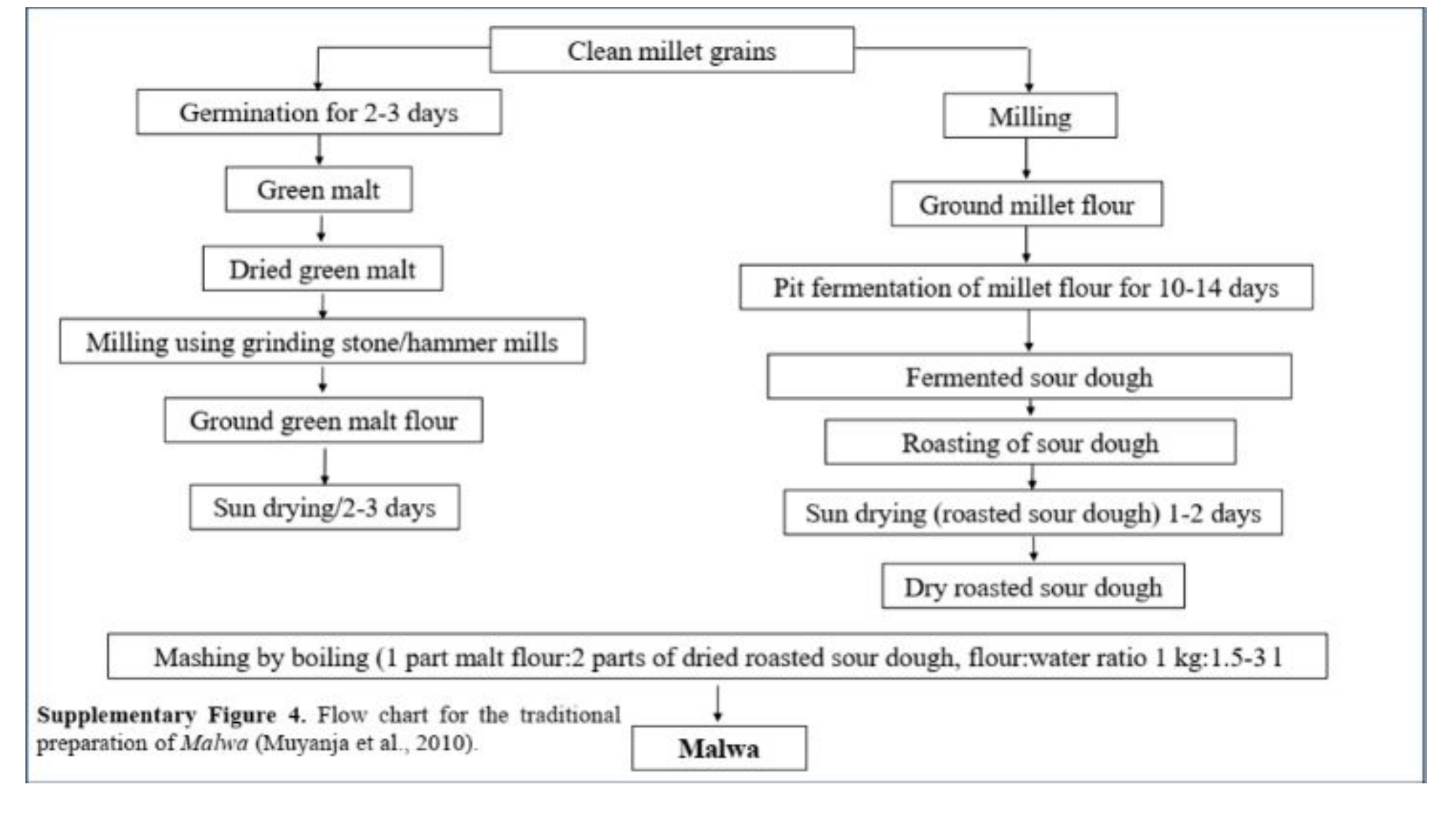

Supplement: Supplementary file 4 — Flow chart for the traditional preparation of Malwa (Muyanja et al., 2010). [file CRF3-24-e70137-s003.pptx]
